# YIV-818-A: a novel therapeutic agent in prostate cancer management through androgen receptor downregulation, glucocorticoid receptor inhibition, epigenetic regulation, and enhancement of apalutamide, darolutamide, and enzalutamide efficacy

**DOI:** 10.3389/fphar.2023.1244655

**Published:** 2023-10-04

**Authors:** Wing Lam, Mohammad Arammash, Wei Cai, Fulan Guan, Zaoli Jiang, Shwu-Huey Liu, Peikwen Cheng, Yung-Chi Cheng

**Affiliations:** ^1^ Department of Pharmacology, Yale University School of Medicine, New Haven, CT, United States; ^2^ School of Pharmaceutical Sciences, Hunan University of Medicine, Huaihua, China; ^3^ Yiviva, Inc, New York, NY, United States

**Keywords:** YIV-818-A, AR, GR, prostate cancer, apalutamide, darolutamide, and enzalutamide

## Abstract

**Introduction:** Prostate cancer is the second leading cause of cancer death among men in the United States. Castration-Resistant Prostate Cancer (CRPC) often develops resistance to androgen deprivation therapy. Resistance in CRPC is often driven by AR variants and glucocorticoid receptor (GR). Thus, drugs that target both could be vital in overcoming resistance.

**Methods:** Utilizing the STAR Drug Discovery Platform, three hundred medicinal plant extracts were examined across 25 signaling pathways to identify potential drug candidates. Effects of the botanical drug YIV-818-A, derived from optimized water extracts of Rubia cordifolia (R.C.), on Dihydrotestosterone (DHT) or Dexamethasone (DEX) induced luciferase activity were assessed in 22RV1 cells harboring the ARE luciferase reporter. Furthermore, the key active compounds in YIV-818-A were identified through activity guided purification. The inhibitory effects of YIV-818-A, RA-V, and RA-VII on AR and GR activities, their impact on AR target genes, and their roles in modifying epigenetic status were investigated. Finally, the synergistic effects of these compounds with established CRPC drugs were evaluated both *in vitro* and *in vivo*.

**Results:** YIV-818-A was found to effectively inhibit DHT or DEX induced luciferase activity in 22RV1 cells. Deoxybouvardin (RA-V) was identified as the key active compound responsible for inhibiting AR and GR activities. Both YIV-818-A and RA-V, along with RA-VII, effectively downregulated AR and AR-V proteins through inhibiting protein synthesis, impacted the expression of AR target genes, and modified the epigenetic status by reducing levels of Bromodomain and Extra-Terminal proteins (Brd2/Brd4) and H3K27Ac. Furthermore, these compounds exhibited synergistic effects with apalutamide, darolutamide, or enzalutamide, and suppressed AR mediated luciferase activity of 22RV1 cells. Co-administration of YIV-818-A and enzalutamide led to a significant reduction of 22RV1 tumor growth in vivo. Different sources of R.C. had variable levels of RA-V, correlating with their potency in AR inhibition.

**Discussion:** YIV-818-A, RA-V, and RA-VII show considerable promise in addressing drug resistance in CRPC by targeting both AR protein and GR function, along with modulation of vital epigenetic markers. Given the established safety profile of YIV-818-A, these findings suggest its potential as a chemopreventive agent and a robust anti-prostate cancer drug.

## Introduction

Prostate cancer is the second leading cause of cancer death among men in the United States. According to the National Cancer Institute over 3 million men in the United States are living with prostate cancer, with 288,300 additional cases and 34,700 deaths expected in 2023. Androgen receptor (AR) signaling plays a key role in development of Benign prostatic hyperplasia (BPH) and prostate cancer (Shiota et al., 2011; Gioeli and Paschal, 2012; Mills, 2014; Wong et al., 2014; Tan et al., 2015). Therefore, androgen or Androgen R targeted therapy has become a major focus for the prevention and treatment of benign prostate hyperplasia and cancer. In addition to surgical interventions, luteinizing hormone-releasing hormone (LHRH) agonist analogues have emerged as a cornerstone of prostate cancer treatment since the mid-1980s. The development of Castration-Resistant Prostate Cancer (CRPC) is inevitable following the long-term treatment of androgen deprivation therapy. For the treatment of metastatic castration-resistant prostate cancer before chemotherapy, in 2011 the FDA approved abiraterone, which targets CYP17A1—a critical enzyme in extragonadal and testicular androgen synthesis. Enzalutamide, which has a higher affinity than first generation antagonists for the AR ligand-binding pocket, was approved by FDA for non-metastatic and metastatic CRPC (Rice et al., 2019). Later on, apalutamide and darolutamide, which have higher selectivity and potency against AR with reductions in brain penetrance, were also approved by FDA for treatment of non-metastatic prostate cancer (Rice et al., 2019).

Many mechanisms for CRPC are proposed and some are responsible for abiraterone resistance and enzalutamide resistance. Resistance mechanisms include altering the metabolic enzymes expression favorable for DHT synthesis, AR gene amplification (Shiota et al., 2011), AR mutation/truncation of LBD (Ligand Binding Domain) (Koivisto et al., 1997; Li et al., 2013). AR splice variants (AR-V) could be commonly (19%–59%) of patients with AR-positive metastatic CRPC (Antonarakis et al., 2014; Lu et al., 2015; Pomerantz et al., 2015). In addition, AR mutations enabling the AR to use other steroid hormones such as glucocorticoids (Buchanan et al., 2001), and the glucocorticoid receptor (GR) replacing AR function (Arora et al., 2013; Shah et al., 2017).

Creating a multi-targeted medication that can simultaneously hinder both the androgen receptor variants and glucocorticoid receptor action holds the potential to overcome drug resistance, extend the duration of treatment efficacy, and ultimately enhance the therapeutic outcomes for prostate cancer patients. Through our STAR (Signal, Transduction, Activity, and Response) Drug Discovery Platform, we studied the effects of three hundred medicinal plant extracts across 25 signaling pathways to identify a drug candidate to target the androgen signaling pathway. YIV-818-A, optimized water extracts of *Rubia cordifolia* (R.C.), was discovered as a novel drug candidate. YIV-818-A, Deoxybouvardin (RA-V), or RA-VII were found to have the ability to effectively target both the androgen receptor and glucocorticoid receptor by down-regulating the androgen receptor protein, impairing the glucocorticoid receptor function, and reducing Brd2/Brd4 levels and H3K27Ac levels, which play a crucial role in AR and GR function. Additionally, the compounds showed synergistic effects with apalutamide, darolutamide, and enzalutamide in inhibiting androgen receptor activity and suppressing the growth of 22RV1 cells. Co-administration of YIV-818-A and enzalutamide also resulted in a marked reduction of 22RV1 tumor growth *in vivo*. In conclusion, YIV-818-A, RA-V, or RA-VII hold promise for overcoming current drug resistance and could be used for the treatment of androgen-dependent disease and castration-resistant prostate cancer (CRPC).

## Materials and methods

### *Rubia Cordifolia* extract preparation methods and standards

YIV-818-A (*Rubia Cordifolia* (batch number, Y1830) water extract spray dried powder) 100 mg/mL was extracted with 1 mL HPLC grade water and heated at 80°C for 30 min. The herbal extract was centrifuged at 12,000 rpm in desktop centrifuge at room temperature for 5 min. The supernatant was transferred into a 2 mL tube and used as a 100 mg/mL. RA-V, RA-VII and RA-XI standard compounds were purchased from Chemfaces.com and were dissolved in DMSO.

### Purification of the active compound with anti-AR activity from *Rubia Cordifolia*

25 g of YIV-818-A was extracted in 100 mL HPLC grade water at 80°C for 30 min. The extract of YIV-818-A was then centrifuged at 10,000 rpm for 10 min and the supernatant was passed through a solid phase column (Millipore-Sigma, Discovery DSC18 10 g). The column was washed sequentially with 50 mL 10% EtOH and 50 mL 30% EtOH and finally 30 mL 50% EtOH was used to elute the fraction containing. The 50% EtOH fraction was vacuum dried and re-dissolved in 3 mL 50% EtOH before passing through a second solid phase column (Millipore-Sigma, Discovery DSC18 10 g). The second column was then washed with 30 mL 0.1% formic acid, 30 mL 10:90 [(Methanol:Acetonitrile 88:12): (0.1% formic acid)], 30 mL 30:70 [(Methanol:Acetonitrile 88:12): (0.1% formic acid)], and 30 mL 45:55 [(Methanol:Acetonitrile 88:12): (0.1% formic acid)]. A fraction was collected by eluting with 30 mL 60:40 [(Methanol:Acetonitrile 88:12): (0.1% formic acid)] and was vacuum dried. Next, the dried material was dissolved and treated with 0.2 N NaOH in a 50% methanol solution (5 mL) for 10 min. The NaOH treated fraction was passed through a C18 column and eluted with AM60 and AM75 as procedure used in the second column. AM75 fraction was then vacuum dried. The dried AM75 fraction was re-dissolved in 3 mL 50% EtOH and then passed through a C18 column (2.5 cm diameter x 50 cm length packed with Discovery DSC18 resin). The C18 column was washed with a gradient of acetonitrile:water from 10% to 80% (100 fractions, 5 mL per each fraction). All collected fractions were then vacuum dried and dissolved in EtOH for the AR luciferase assay and Western blotting. The fractions which successfully inhibited the AR activity and downregulate the AR and CycD1 protein of 22RV1 cells were subjected to UHPLC Q-Exactive Orbitrap MS for analysis (Thermo Fisher Scientific, Bremen, Germany). The fraction with the purified active compound with [M+H]^+^ *m/z* = 757.3549 (positive mode) was dissolved in CDCl_3_ and subjected to NMR analysis (Bruker Daltonics, Bremen, Germany) for chemical structure determination.

### UHPLC Q-Exactive Orbitrap MS condition

A Dionex Ultimate 3000 UHPLC and the Q-Exactive Focus Orbitrap MS was connected via an electrospray ionization (ESI) source. The fractions were performed on a Thermo Scientifific Hypersil GOLD™ aQ (100 mm × 2.1 mm, 1.9 mm) at the flow rate of 0.3 mL/min. The mobile phases consisted of 0.1% formic acid aqueous solution (solvent A) and acetonitrile (solvent B) with the following gradient program: 0–2 min, 95%–90% A; 2–5 min, 90%–80% A; 5–10 min, 80%–75% A; 10–12 min, 75%–45% A; 12–20 min, 45%–20% A; 20–25 min, 20%–5% A; 25–26 min, 5%–95% A; 26–30 min, 95% A. The sample injection volume was 2 µL. The key mass spectrometer was running at positive mode with the mass range at *m/z* 120–1,000. The key parameters were as follows: spray voltage, 3.5 kV (+); the sheath gas flow rate, 35 arb; aux gas flow rate, 10 arb; capillary temperature, 320°C; heater temperature, 350°C; resolution, 70,000. Data acquisition and processing were carried out with the Xcalibur version 4.2.

### Prostate cancer cell culturing methods

22RV1 and LNCaP prostate cancer cells were grown in corning T75 cell culture flasks in RPMI1640 media supplemented with 5% FBS, 50 μg/mL Kanamycin in a 37°C, 5% CO_2_ incubator.

### Establishment of PSA luciferase reporter in 22RV1, LNCaP and PC3 cells and overexpression of GR in LNCaP cells

The 22RV1, LNCaP and PC3 cell lines were stably transfected with a PGL4.2 luciferase reporter (Promega, Madison, USA). This reporter contains a sequence (3xgtaattgcagaacagcaagtgctagctctc) representing the wild type of Androgen Response Element (ARE) derived from the Prostate-Specific Antigen (PSA) promoter. As a control, cells were also transfected with the PGL4.2 luciferase reporter that does not include the PSA promoter. This transfection was accomplished using Lipofectamine 3000 (Thermo Fisher Scientific, Waltham, MA). Stable clones were selected and maintained with puromycin (0.5 μg/mL). DHT (25 nM) and DEX (50 nM) was used to stimulate androgen receptor activity for a 24-hour period. The pcDNA5 plasmid (Thermo Fisher Scientific, Waltham, MA), carrying the full open reading frame of GR, was transfected into LNCaP cells, and hygromycin (50 μg/mL) was employed to select and maintain stable clones.

### Luciferase assay

10^4^ reporter cells per well of 96-well plate were seeded in RPMI1640 media supplemented with 5% dialysis activated carbon treated FBS, 50 μg/mL Kanamycin in a 37°C, 5% CO2 incubator for 48 h. Cells were treated with herbal extracts at 30, 100, 300, and 600 μg/mL for 24 h in a 37°C-CO_2_ incubator. DHT (25 nM) was used to stimulate androgen receptor activity. Dexamethasone (50 nM) was used to stimulate glucocorticoid receptor activity. Cells were lysed using luciferase lysis buffer after which luciferase buffer with luciferin was added to generate luminescence. Luminescence was recorded using a luminescence microplate reader. The IC_50_ is the concentration of treatment agent required to reduce the measured luminescence to half of its maximal value. IC_50_ was determined by the median-effect equation of the Chou-Talalay method (Chou and Talalay, 1977).

### Drug-drug interaction analysis

Isobologram plots based on combination index (Chou and Talalay, 1977) were used to determine additive, synergistic, and antagonistic interactions of the combinations. Isobologram plots: (D)1/(Dχ)1 against (D)2/(Dχ)2 in which (Dχ)1 and (Dχ)2 represented concentrations of each drug alone to exert 50% inhibition, while (D)1 and (D)2 were concentrations of drugs in combination to achieve 50% inhibition. Different ratio of two herbs were used to determine their combination effects. Additive interaction: Points on the diagonal line. Synergistic interaction: Points below the diagonal line. Antagonistic interaction: Points above the diagonal line.

### Western blotting

Cell lysis was performed using 2x SDS sample buffer (62.5 mM Tris-HCl, 2% SDS, 10% glycerol, 50 mM DTT, and 0.05% bromophenol blue). Cell lysates were sonicated for 10 s to shear DNA. Cell extracts were then electrophoresed through 10% SDS-polyacrylamide gels and transferred to 0.2 µm nitrocellulose membranes (Bio-Rad Laboratories, Hercules, CA) with a Miniprotein II transferring apparatus (Bio-Rad). The non-specific interaction of membrane was blocked with 5% non-fat milk and probed in TBS-T buffer (1x TBS buffer, 0.2% Tween 20) containing. Monoclonal rabbit anti-AR (1:5000), was used to detect the androgen receptor (Abcam #133273), Glucocorticoid Receptor (D8H2) XP® Rabbit mAb #3660, ERα Antibody (F-10): sc-8002, BRD4 (E2A7X) Rabbit mAb #13440, Brd2 (D89B4) Rabbit mAb #5848, PARP (46D11) Rabbit mAb #9532, Acetyl-Histone H3 (Lys27) (D5E4) XP® Rabbit mAb #8173 and a monoclonal actin antibody diluted 1:2500 (Sigma, St. Louis, MO) was used to detect β-actin as the internal control to normalize protein loading. The membranes were then incubated with horseradish peroxidase-conjugated anti-mouse IgG and anti-rabbit IgG (1: 5,000; Sigma). Enhanced chemiluminescence reagents (Perkin-Elmer Life Science Products, Boston, MA) were used to visualize the immunoreactive bands and the densities of protein bands were scanned analyzed using ImageJ software from the NIH.

### Real time quantitative PCR (RT-qPCR) for mRNA expression

RNA was extracted from herb treated cells using the Roche High Pure RNA isolation kit. cDNA was then generated from RNA samples using a Bio-rad iScript Advanced cDNA synthesis kit for RT-qPCR. qPCR was performed using human AR, KLK2, PSA, CycD1 and β-actin primers (Table 1) and iTaq™ Universal SYBR® Green Supermix in a CFX PCR machine (Bio-rad). Duplicate well were performed for each sample, and the entire experiment was repeated three times Relative mRNA expression was calculated based on the change of the threshold cycle relative to the internal control, β-actin, using a standard curve generated by purified PCR products.

**TABLE 1:** Primer sequences for qRT-PCR analysis.

Gene	Forward/Reverse	DNA sequence
Human AR	F1	CCT​GGC​TTC​CGC​AAC​TTA​CAC
	R1	GGA​CTT​GTG​CAT​GCG​GTA​CTC​A
Human KLK2	F1	GGT​GGC​TGT​GTA​CAG​TCA​TGG​AT
	R1	TGT​CTT​CAG​GCT​CAA​ACA​GGT​TG
Human PSA	F1	ACC​AGA​GGA​GTT​CTT​GAC​CCC​AAA
	R1	CCC​CAG​AAT​CAC​CCG​AGC​AG
Human CycD1	F1	CCA​TCC​AGT​GGA​GGT​TTG​TC
	R1	AGC​GTA​TCG​TAG​GAG​TGG​GA
Human β-actin	F1	GCC​ACG​GCT​GCT​TCC​AGC​TCC
	R1	TTG​TGC​TGG​GTG​CCA​GGG​CAG​TGA

### Animal studies

22RV1 cells (2 × 10^6^ cells in 100 μL Matrigel, BD Biosciences) were transplanted subcutaneously into 8-week-old female NCR-nude mice (Taconic Biosciences, Inc., Rensselaer, NY). Body weight, tumor size, and mortality of the mice were monitored daily. After 10–14 days, mice with tumor sizes of 180 mm^3^ were selected. Tumor volume was examined by using the formula length × width^2^ × π/6. Each group consisted of five mice. YIV-818-A was administered orally for 7 days (500 mg/kg po, BID) and/or enzalutamide was administered orally for 7 days (5 mg/kg po, QD). In the control groups, mice were administered water orally. All animal experiments were carried out in accordance with the relevant guidelines and regulations approved Yale University Institutional Animal Care and Use Committee (IACUC) protocol. Animal experimental protocols were approved by Yale University Institutional Animal Care and Use Committee (IACUC). Animal studies were carried out in compliance with the ARRIVE guidelines.

### Detection of PSA protein of plasma of animal

PSA (Total)/KLK3 Human ELISA Kit (Thermofisher Scietific) was used to detect PSA protein of plasma collected from animal using PST tube with heparin (BD biosciences) according to the manual provided in the kit.

### Statistical analysis

Two-way ANOVA analysis (GraphPad Prism 9) was employed to discern differences between two data curves. Additionally, t-tests (Microsoft Excel) were conducted to ascertain statistical significance between group comparisons. Differences were considered statistically significant at *p* < 0.05.

## Results

### Deoxybouvardin (RA-V) is the active compound of *Rubia cordifolia* responsible for inhibiting AR activity and downregulating AR protein

YIV-818A water extract was fractionized using C18 columns with different gradients of methanol and acetonitrile (please see detail in materials and methods). The eluted fractions (F9 to F15) from the final C18 column had a relatively strong inhibitory effect on AR driven luciferase activity and could also downregulate AR/AR-V (AR splice variants) protein of 22RV1 (Supplementary Figures S1A, B). Fraction 11 had a single peak (highest intensity amount of F9 to F15) with [M+H]^+^ *m/z* = 757.3549 that was detected using high resolution LC-MS (Supplementary Figure S1C). By marching to chemical database, [M+H]^+^; calcd for Deoxybouvardin (RA-V) C_40_H_49_N_6_O_9_ is 757.3555 which is closest to our detected mass (*m/z* = 757.3549) of the purified compound. RA-V was purchased from a commercial source and was subjected to LCMS. Both chemicals of F11 and RA-V had some retention time with [M+H]^+^ *m/z* = 757.3549 (Supplementary Figure S1C). They also had the same mass spectrum [M+H]^+^ *m/z* = 757.3549, [M+Na]^+^ *m/z* = 779.3364 (Supplementary Figure S1D). In addition, ^1^H-NMR and ^13^C-NMR analysis confirmed the chemical of F11 (dissolved in CDCl_3_) had same chemical shifts with Deoxybouvardin (RA-V) (Supplementary Table S1) as previously reported (Itokawa et al., 1983). Please see other NMR spectrum including ^1^H, ^13^C, DEPT90, DEPT135, COSY, HMBC, HSQC, NOESY in Supplementary Data for NMR analysis.

### YIV-818-A, RA-VII and RA-V could inhibit both DHT and DEX induced luciferase activity of prostate cancer cells

**FIGURE 1:**
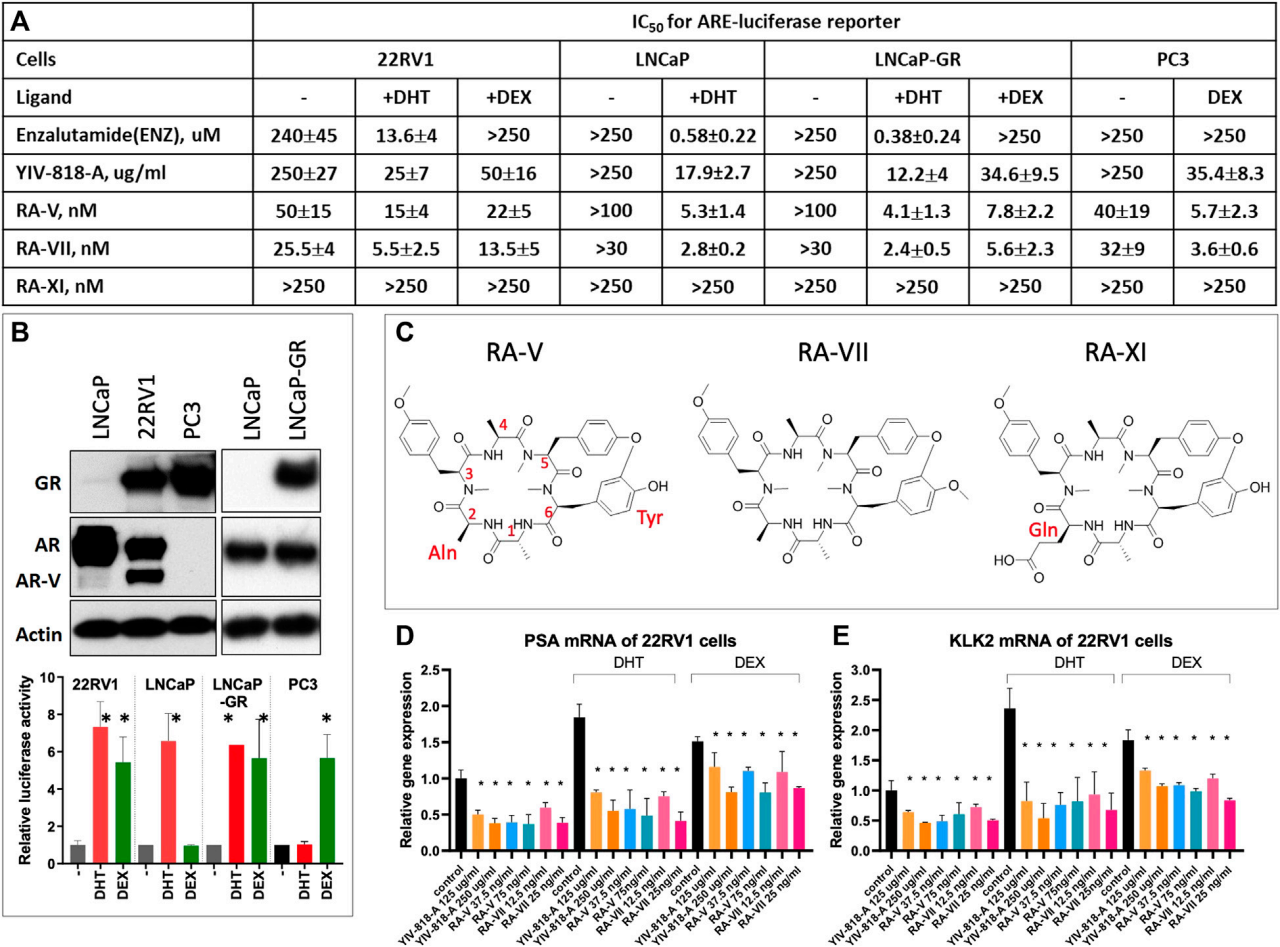
Comparing the potencies of enzalutamide, YIV-818-A, RA-V, RA-VII and RA-XI on LNCaP and 22RV1 AR luciferase reporter cells. **(A)** Effect of YIV-818-A, RA-V, RA-VII, RA-XI, and enzalutamide on AR luciferase reporter activity of 22RV1 cells without or with DHT or with DEX and LNCAP cells without or with DHT. LNCaP cells and 22RV1 cells, carrying AR-luciferase reporter, were treated with YIV-818-A, RA-V, RA-VII, RA-XI and enzalutamide in absence or presence of DHT or DEX for 24 h. Values are IC_50_ (dose of herbal extract or compound required for 50% inhibition of the luciferase activity as comparing to control). **(B)** Western blotting for the protein expression of GR, AR, AR-V (AR splice variants) and actin (upper panel) and luciferase activity with or without DHT or DEX (lower panel) of 22RV1, LNCaP, LNCaP-GR and PC3 cells and. GAPDH was used as protein loading control for normalization. **(C)** Chemical structures of RA-V, RA-VII and RA-XI. RT-qPCR analysis for the effect of YIV-818-A, RA-V, RA-VII on mRNA expression of PSA **(D)** and KLK2 **(E)** of 22RV1 cells with or without DHT and DEX. *p*-value (*t*-test) less than 0.05 (Control with or with DHT or DEX vs. Treatment) was highlighted with (*) in the figure. Details of experimental procedures are given in *Materials and methods*.

In Figure 1A, the presence of DHT resulted in a remarkable 23-fold increase in enzalutamide resistance in the castration-resistant prostate cancer cell line, 22RV1 (Sramkoski et al., 1999), 1999), compared to the IC_50_ observed in LNCaP cells. This finding is consistent with previous studies (Li et al., 2013; Nakazawa et al., 2014) suggesting that AR splice variants in 22RV1 cells (Figure 1B), which possess a truncated ligand-binding domain, maintain continuous activity and are unresponsive to enzalutamide binding. RA-VII and RA-XI are both hexapeptide analogs of RA-V (Figure 1C). RA-XI was detected in YIV-818-A and other batches of RC, while RA-VII was barely detected in any batch of RC. Intriguingly, up to 250 nM of RA-XI did not inhibit luciferase activity in any assay, whereas RA-V, similar to YIV-818-A, effectively inhibited both DHT- and Dex-induced luciferase activity in both LNCaP and 22RV1 cells (Figure 1A). Furthermore, RA-VII exhibited higher potency than RA-V in all luciferase assays (Figure 1A).

YIV-818A demonstrated enhanced inhibitory action in 22RV1 cells in the presence of DHT, suggesting a potential heightened susceptibility of wild type AR to YIV-818A in these cells (Figure 1A). Notably, both RA-V and RA-VII showed only slight differences in IC_50_ values with or without DHT in 22RV1 cells, indicating their enhanced targeting proficiency for both AR and AR-V (AR splice variants) (Figure 1A). The above results indicate that the presence of DHT can significantly impact enzalutamide resistance in 22RV1 cells and support the notion that YIV-818A, RA-V, and RA-VII effectively inhibit AR and AR-V (AR splice variants) activity in prostate cancer cells. The IC_50_ values for YIV-818-A, RA-V, and RA-VII in 22RV1 cells were discerned to be 1 to 3 times higher than those in LNCaP cells under DHT influence (Figure 1A). Even though YIV-818-A, RA-V, and RA-VII may not completely counteract the drug resistance portrayed by AR-V (AR splice variants) bearing 22RV1 cells, they exhibited IC_50_ values significantly bridging the gap between LNCaP and 22RV1 cells, unlike enzalutamide. This represents a substantial advancement over the striking 23-fold IC_50_ disparity observed with enzalutamide. Consequently, our data suggests that YIV-818-A, RA-V, and RA-VII may possess potential to target prostate tumors bearing either wild type AR or AR-V (AR splice variants), thereby promising to substantially attenuate, if not completely nullify, the drug resistance manifested in 22RV1 cells.

Additionally, it has been reported that around 30% of enzalutamide-resistant patients exhibit overexpression of the glucocorticoid receptor (GR), which can substitute for AR function and activate downstream targets of the androgen receptor. In our study, we observed that enzalutamide was unable to inhibit GR-mediated luciferase activity in the AR luciferase reporter assay using 22RV1 cells that express GR (Figure 1A). In contrast, YIV-818-A effectively suppressed dexamethasone (Dex)-induced luciferase activity in 22RV1 cells. To further investigate the inhibitory effects of YIV-818A, RA-V, and RA-VII on GR-driven activity, we compared the actions of Enzalutamide, YIV-818A RA-V, and RA-VII in three different cell lines: LNCaP cells that only express AR and respond solely to DHT, LNCaP-GR cells that express both AR and GR and respond to both DHT and DEX, and PC3 cells that only express GR and respond solely to DEX (Figure 1B). As shown in Figure 1A, enzalutamide could only inhibit DHT-induced luciferase activity in LNCaP and LNCaP-GR cells but had no effect on DEX-induced luciferase activity in LNCaP-GR or PC3 cells. In contrast, YIV-818A RA-V and RA-VII were able to inhibit DEX-induced luciferase activity in LNCaP-GR or PC3 cells (Figure 1A). In conclusion, our findings demonstrate that YIV-818A RA-V and RA-VII could effectively inhibit both DHT-driven AR activity and DEX-driven GR activity in prostate cancer cells.

In addition to luciferase reporter assays, we further confirmed that YIV-818-A, RA-V and RA-VII could inhibit DHT or DEX triggered endogenous AR target gens; *PSA* and *KLK2* of 22RV1 cells by using qRT-PCR (Figures 1D, E).

Overall, our findings suggest that YIV-818-A, RA-V, and RA-VII hold promise as potential treatments for prostate cancers with AR, AR-V (AR splice variants) and might be beneficial in enzalutamide-resistant cases with GR overexpression.

### YIV-818-A, RA-V and RA-VII could downregulate AR, AR-V (AR splice variants), CycD1 protein through inhibiting protein synthesis

**FIGURE 2:**
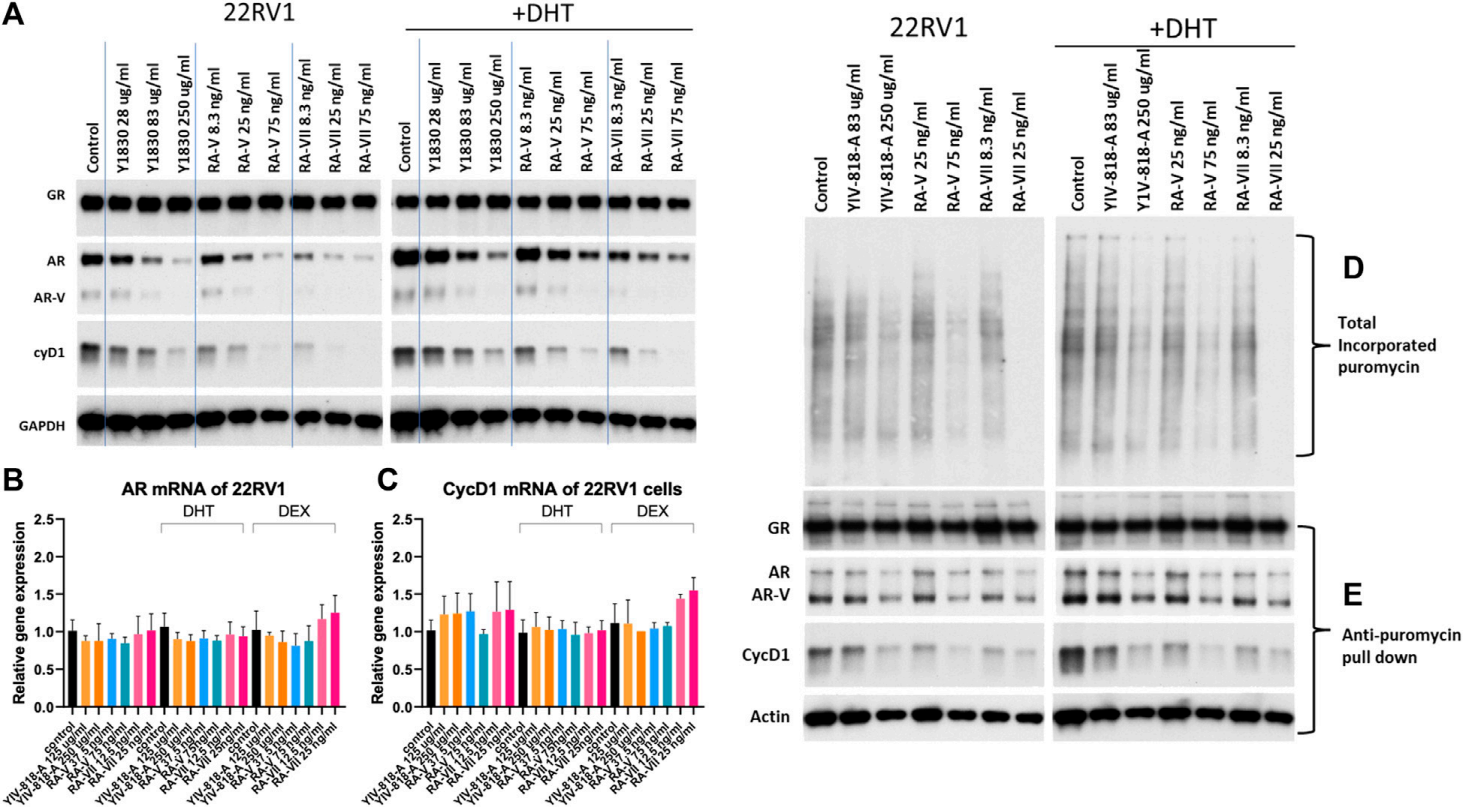
Effect of YIV-818-A, RA-V or RA-VII on GR, AR, AR-V (AR splice variants) and CycD1 protein expression and AR and CycD1 mRNA expression of 22RV1 cells as. **(A)** Western blotting for protein expression of GR, AR, AR-V (AR splice variants) and CycD1 of 22RV1 following treatment of YIV-818-A, RA-V or RA-VII without or with DHT (25 nM) for 24 h. Specific antibodies were used to determine the protein expression in Western blotting. GAPDH was used as protein loading control for normalization. 22RV1 cells were treated with YIV-818-A, RA-V or RA-VII for 24 h without or with DHT. Total RNA was extracted for RT-qPCR using specific primers for AR**(B)** and CycD1**(C)**. β-actin mRNA was used for normalization. All results were normalized to no ligand control. **(D)** Western blotting for detection of *de novo* synthesis polypeptides of 22RV1 cells. Puromycin (1uM) was used to label newly translated polypeptides of 22RV1 cells in presence of YIV-818-A, RA-V or RA-VII without and with DHT (25 nM) for 45 min. Monoclonal Anti-puromycin antibody was used to detect puromycin incorporated polypeptides. **(E)** Western blotting for detection of newly translated AR, AR-V (AR splice variants) and CycD1. After puromycin labeling, 22RV1 cells were lysed in IP buffers. Anti-puromycin antibody and protein-A/G magnetic beads were used to pull down newly synthesis polypeptides and then specific antibodies for AR, CycD1 and Actin were used to detect pull down AR, AR-V (AR splice variants), CycD1 and Actin **(E)**. Details of experimental procedures are given in *Materials and methods*.

Based on the LC-MS analysis, it was determined that the water extract of YIV-818-A at 250 μg/mL contained approximately 75 ng/mL of RA-V. As a result, we utilized a concentration range of YIV-818-A spanning from 28 μg/mL to 250 μg/mL, and a concentration range of RA-V from 8.3 ng/mL to 75 ng/mL, to treat 22RV1 cells with or without DHT for a duration of 24 h (Figure 2A). These specific concentration ranges were chosen to ensure effective exposure of the cells to YIV-818-A and RA-V while considering their respective potencies (Figure 2A). As depicted in Figure 2A, RA-V exhibited similar potency to YIV-818-A in downregulating AR (full-length), AR-V (AR splice variants), and cyclin D1 protein in 22RV1 cells, both with and without DHT. Based on these results, RA-V appears to be the key compound in YIV-818-A responsible for downregulating AR, AR-V (AR splice variants), and cyclin D1 in 22RV1 cells. In comparison, RA-VII required 25 ng/mL to achieve similar effects as those produced by 75 ng/mL of RA-V (Figure 2A). Consequently, RA-VII demonstrated approximately three times greater potency than RA-V in downregulating AR, AR-V (AR splice variants), and cyclin D1 in 22RV1 cells. YIV-818-A, RA-V, or RA-VII were also capable of downregulating AR and cyclin D1 protein in LNCaP cells (Supplementary Figure S2). YIV-818-A, RA-V, or RA-VII had no effect on GR protein expression and GR nuclear localization in 22RV1 cells in the presence of dexamethasone (dex) (Supplementary Figures S3A, B). These findings suggest that YIV-818-A, RA-V, or RA-VII might exhibit selectivity in downregulating certain hormone receptor proteins.

In Figures 2B, C, YIV-818-A, RA-V, or RA-VII did not significantly impact AR and cyclin D1 mRNA expression in 22RV1 cells. This suggests that the downregulation of AR and cyclin D1 protein by YIV-818-A, RA-V, or RA-VII is not mediated through mRNA regulation. Since Bouvardin and SVC112, analogs of RA-V and RA-VII, have previously been identified as protein translation inhibitors that can inhibit *de novo* protein synthesis (Zalacain et al., 1982; Chan et al., 2004; Keysar et al., 2020), we investigated whether YIV-818-A, RA-V, or RA-VII could inhibit AR by suppressing its protein synthesis. In Figure 2D and Supplementary Figure S4, we demonstrated that YIV-818-A, RA-V, or RA-VII could inhibit the incorporation of puromycin into newly synthesized polypeptides of 22RV1 cells. When puromycin-labeled polypeptides were pulled down for Western blot analysis, the levels of newly synthesized AR, AR-V (AR splice variants), and cyclin D1, but not GR and actin, were decreased by YIV-818-A, RA-V, or RA-VII treatments (Figure 2E). In conjunction with the above results, we provide evidence that YIV-818-A, RA-V, or RA-VII downregulate AR, AR-V (AR splice variants), and cyclin D1 primarily through protein synthesis inhibition, rather than mRNA regulation.

### YIV-818-A, RA-V and RA-VII could affect epigenetic status by inhibiting BRD protein expression and H3K27 acetylation

**FIGURE 3:**
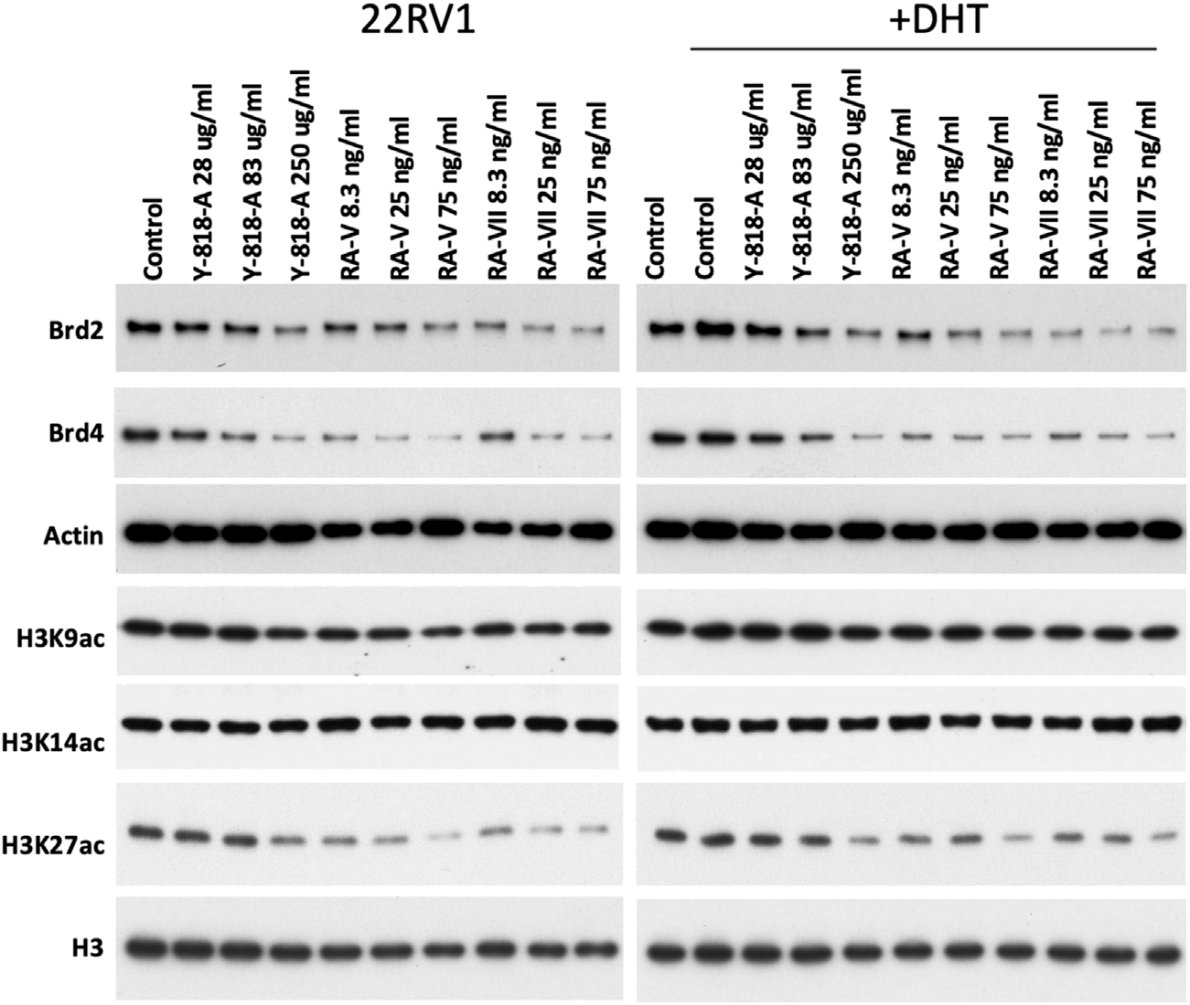
Effects of YIV-818-A, RA-V or RA-VII on protein expression of Brd2, Brd4 and histone acetylation at lysine 9, 14 and 27 of 22RV1 cells without or with DHT. Western blotting for protein expression of Brd2, Brd4, H3K9ac, H3K14ac, H3K27ac of 22RV1 following treatment of YIV-818-A, RA-V and RA-VII without or with DHT (25 nM) for 24 h. Specific antibodies were used to determine the protein expression in Western blotting. Actin and Histone 3 (H3) was used as protein loading control for normalization. Details of experimental procedures are given in *Materials and methods*.

The BET (Bromodomain and Extra-Terminal Domain) subfamily of bromodomain proteins plays a crucial role in AR and GR-dependent enhancer activation, leading to the transcription of target genes regulated by these receptors (Asangani et al., 2014; Nagarajan et al., 2014; Shah et al., 2017). By recognizing acetylated histones (H3K27Ac) on enhancer regions, BET proteins facilitate the recruitment of AR or GR transcriptional machinery, leading to the activation of target gene expression and the promotion of cell-specific responses to androgens and glucocorticoids (Asangani et al., 2014; Nagarajan et al., 2014; Shah et al., 2017). BET inhibitors have been demonstrated to suppress breast and prostate cancer cell growth. Treatment with YIV-818-A, RA-V, or RA-VII led to a decrease in Brd2 and Brd4 protein expression in 22RV1 cells, both with and without DHT (Figure 3). Moreover, YIV-818-A, RA-V, or RA-VII reduced histone 3 lysine 27 acetylation (H3K27Ac), which is required for BRD binding at enhancers, but did not affect H3K9Ac or K14Ac (Figure 3).

These findings suggest that YIV-818-A, RA-V, or RA-VII might reduce the binding of Brd proteins to promoter/enhancer regions of AR and GR target genes, thereby impacting their transcription. Additionally, the downregulation of Brd proteins and H3K27Ac could represent one mechanism of action through which YIV-818-A, RA-V, or RA-VII inhibit GR transcriptional activity. These results also imply that YIV-818-A, RA-V, or RA-VII are not pan-inhibitors for histone acetyltransferases (HAT), but rather may selectively inhibit the transcription of a subset of genes that are dependent on H3K27Ac.

### YIV-818-A, RA-V or RA-VII exhibit synergistic inhibition of AR activity in 22RV1 cells and LNCaP cells upon combination with enzalutamide, apalutamide, and darolutamide

Given that YIV-818-A, RA-V, or RA-VII exhibit distinct mechanisms of action in inhibiting AR compared to the FDA-approved androgen inhibitors, enzalutamide, apalutamide, and darolutamide used in prostate cancer treatments, we speculated that YIV-818-A, RA-V, or RA-VII might have the potential to synergistically enhance the therapeutic effects of enzalutamide, apalutamide, or darolutamide.

**FIGURE 4:**
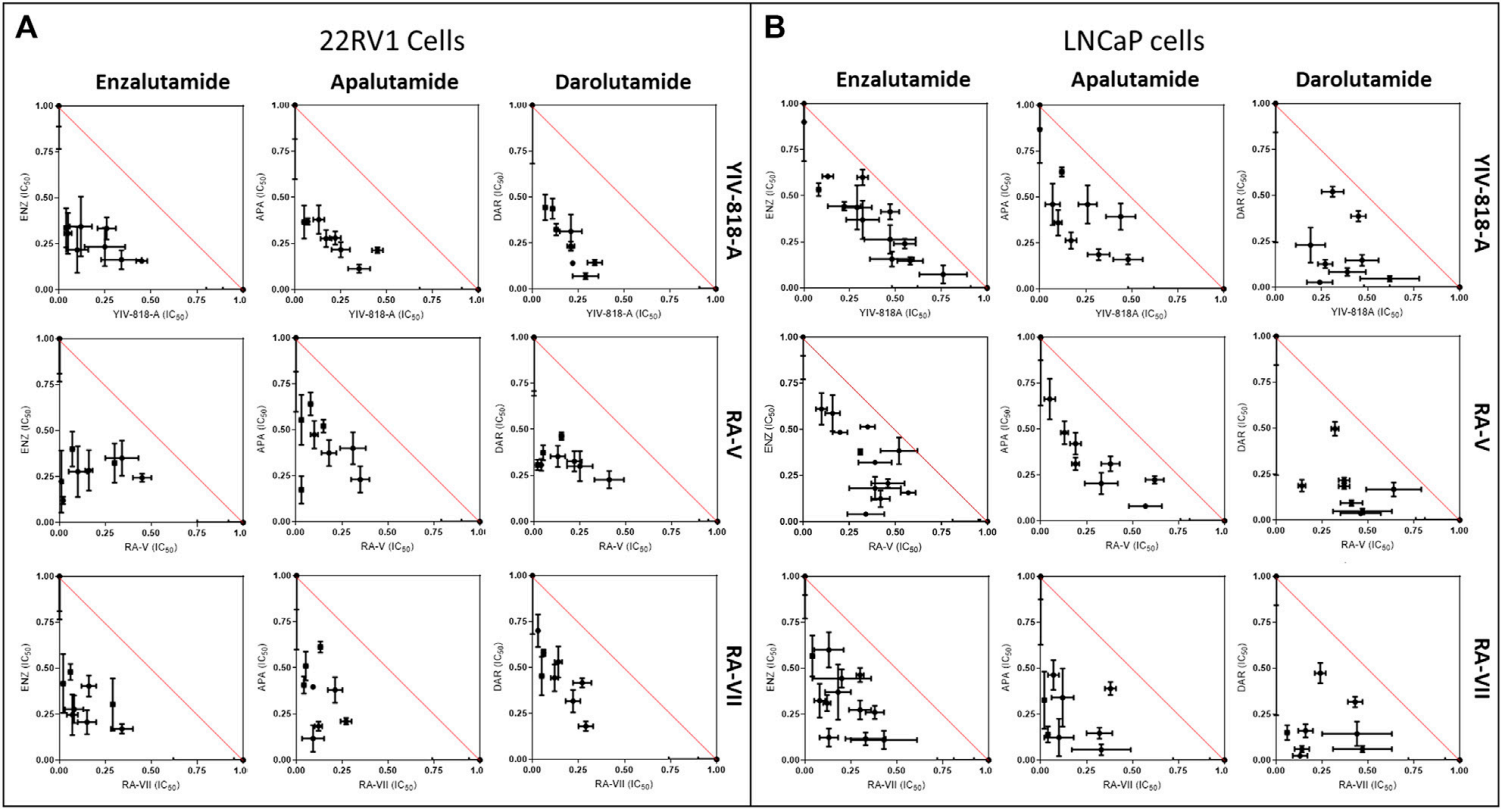
Interaction of YIV-818-A, RA-V or RA-VII with enzalutamide (ENZ), apalutamide (APA) or darolutamide (DAR) on inhibiting DHT induced AR mediated luciferase activity of 22RV1 cells **(A)** and LNCaP cells **(B)**. Isobologram plots were used to determine additive, synergistic, and antagonistic interactions of the combinations. Additive interaction: Points on the red-diagonal line. Synergistic interaction: Points below the red-diagonal line. Antagonistic interaction: Points above the red-diagonal line. Details of experimental procedures are given in *Materials and methods*.

To assess drug interactions between YIV-818-A, RA-V, or RA-VII and the current drugs, we performed an isobologram analysis based on the combination index. Our results demonstrated that combinations of YIV-818-A, RA-V, or RA-VII with enzalutamide, apalutamide, or darolutamide exhibited synergistic effects (most data points from all treatments fell below the red-diagonal line of the isobologram plots) in inhibiting DHT-driven AR activity in both 22RV1 and LNCaP cells (Figures 4A, B). Therefore, YIV-818-A, RA-V, or RA-VII showed potential to enhance the efficacy of enzalutamide, apalutamide, or darolutamide in the treatment of prostate cancers.

YIV-818-A enhanced enzalutamide action against 22RV1 tumor growth *in vivo*. The compelling *in vitro* evidence of synergistic AR activity inhibition by YIV-818-A and enzalutamide led us to explore their combined efficacy in an *in vivo* setting. We proceeded to validate this hypothesis using a 22RV1 xenograft model in nude mice.

**FIGURE 5:**
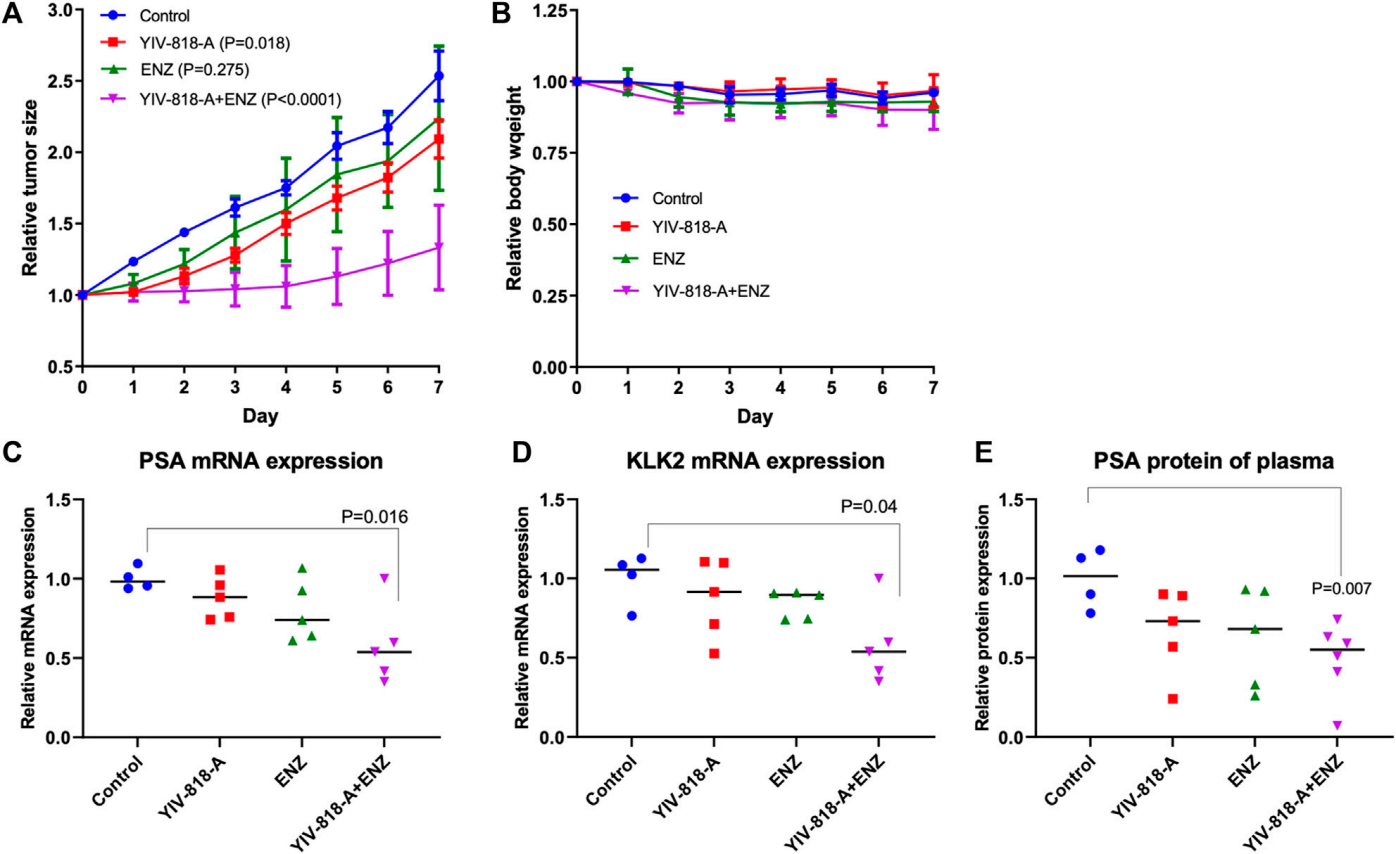
Effect of YIV-818-A and/or enzalutamide on 22RV1 tumor growth of NCR-nude mice. **(A)** Effect of YIV-818-A (500 mg/kg, PO, BID) and/or enzalutamide (5 mg/kg, PO, QD) on 22RV1 tumor growth of NCR-nude mice. **(B)** Effect of YIV-818-A and/or enzalutamide on animal body weight during the treatments. qRT-PCR analysis for PSA mRNA **(C)** and KL2 mRNA **(D)** of 22RV1 tumor following the treatments. **(E)** PSA protein detection of plasma of animal following the treatments. ELISA was used to detect PSA protein of plasma. Two-way ANOVA analysis was employed to discern differences between two data curves. Additionally, t-tests were conducted to ascertain statistical significance between group comparisons. Details of experimental procedures are given in *Materials and Methods*.

Oral administration of YIV-818-A (500 mg/kg, twice daily) resulted in a significant deceleration of tumor growth (*p* = 0.018). On the other hand, enzalutamide (5 mg/kg, once daily) was not found to substantially decrease the growth of 22RV1 tumors in the nude mice (Figure 5A). However, a strikingly pronounced inhibition of 22RV1 tumor growth (*p* < 0.0001) was observed when YIV-818-A and enzalutamide were co-administered (Figure 5A). It is important to note that the treatments with YIV-818-A and/or enzalutamide had no discernible impact on the body weight of the test subjects (Figure 5B). In a deeper examination of the tumor tissue, we observed a considerable decrease in the mRNA expression levels of PSA and KLK2 - known target genes of AR - in response to the combined therapy of YIV-818-A and enzalutamide (Figures 5C, D). Furthermore, we also noted a reduction in PSA protein levels in plasma following this combined treatment (*p* < 0.007) (Figure 5E).

These results robustly align with our *in vitro* findings and indicate that a combined therapeutic approach with YIV-818-A and enzalutamide could offer an enhanced strategy to inhibit the growth of prostate cancer.

### RA-V can serve as a quality control marker to select *Rubia cordifolia*

**FIGURE 6:**
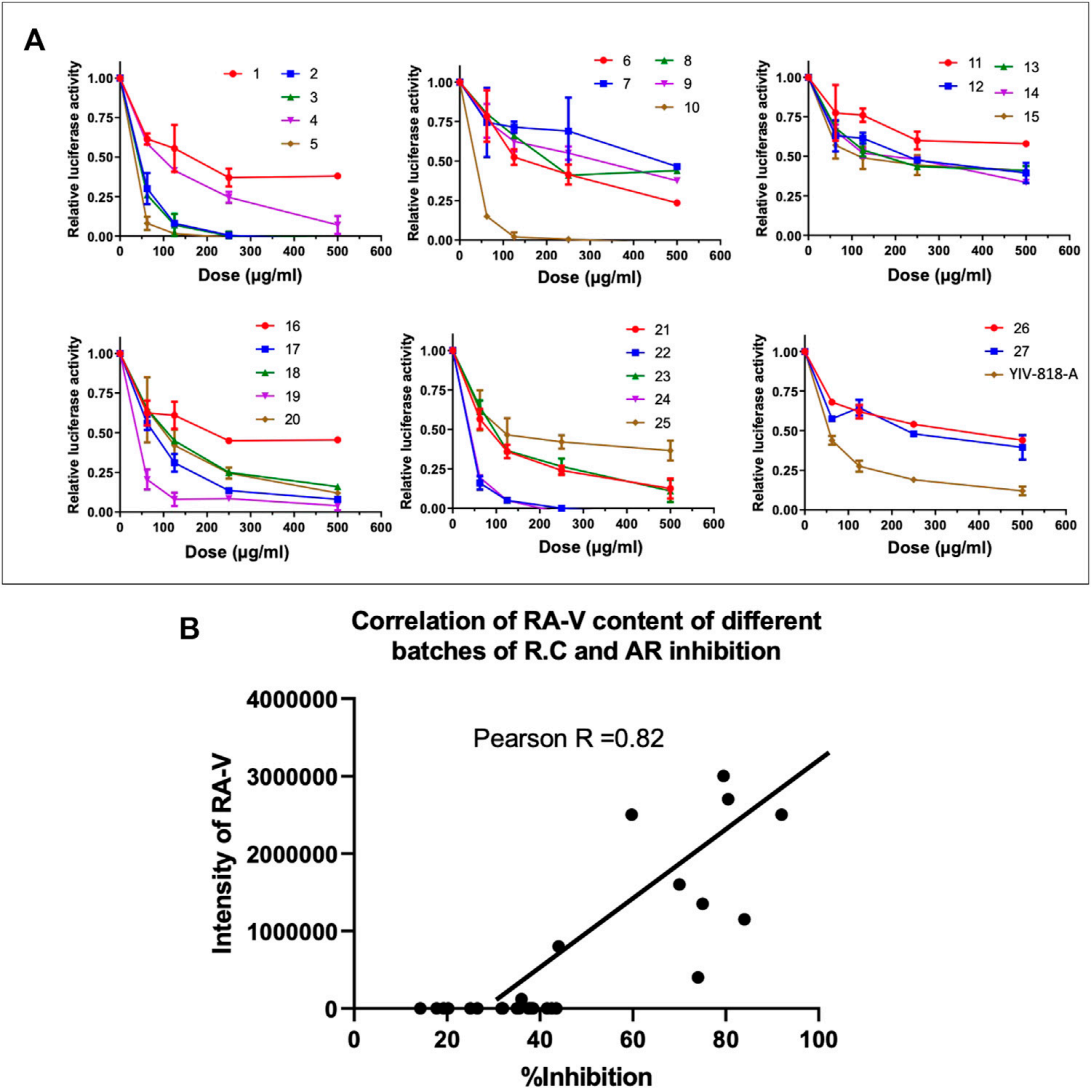
Correlation between RA-V of *Rubia cordifolia* (R.C.) and AR Inhibition in 22RV1 Cells. **(A)** Effect of different batches of RC on AR driven luciferase activity of 22RV1 in presence of DHT. **(B)** Pearson correlation analysis for the content of RA-V of different batches of *Rubia cordifolia* (R.C.) and AR inhibition (%). Details of experimental procedures are given in *Materials and Methods*.

27 different batches of *Rubia cordifolia* (R.C.) and YIV-818-A water extracts were used to treat 22RV1-AR-luciferase reporter cells in present of DHT for 24 h. Results indicated that different batches of *Rubia cordifolia* (R.C.) had different potency in inhibiting DHT triggered AR activity (Figure 6A). The RA-V content of different batches was measured using LCMS. The content of RA-V of different batches was correlated to AR inhibition (%) using Pearson correlation. Results indicated that the content of RA-V of *Rubia cordifolia* (R.C.) had some degree of correlation to the AR inhibition (%) (R = 0.819) (Figure 6B). Therefore, the RA-V content of a specific batch of *Rubia cordifolia* (R.C.) could be used to predict its potency of AR inhibition. An increased content of RA-V in the batches of *Rubia cordifolia* (R.C.) increased the likelihood of stronger AR activity inhibition.

## Discussion

Enzalutamide, apalutamide, and darolutamide are second-generation androgen receptor (AR) blockers approved for both non-metastatic and metastatic castration-resistant prostate cancer (CRPC) (Cattrini et al., 2022). These drugs significantly extend metastasis-free survival to about 36–40 months, compared to 14.7–18.4 months in the placebo groups (Cattrini et al., 2022). However, grade 3 adverse events or higher occur in approximately 25% of patients, with fatigue, hypertension, diarrhea, and weight loss being the most common side effects. Furthermore, enzalutamide carries a risk of causing seizure activity (Cattrini et al., 2022). Drug resistance, often manifested through AR splice variants, mutations, or glucocorticoid receptor (GR) substitution of AR function, is a common cause of treatment failure (Rice et al., 2019).

In this study, we present YIV-818-A and its active component, RA-V, as novel compounds capable of inhibiting both AR and GR activity. Similarly, RA-VII, a bicyclic hexapeptide analog of RA-V, exhibits comparable activity. Our Western blotting results reveal that YIV-818-A, RA-V, or RA-VII can reduce AR protein levels and diminish mRNA expression of AR target genes such as PSA and KLK2. Previous research demonstrated that SVC112, an analog of RA-VII, selectively reduces Cyclin D1 (CycD1) protein levels by obstructing protein translation in Head and Neck Squamous Carcinoma (Keysar et al., 2020). Furthermore, RA-V has been found to impede protein translational elongation by inhibiting the connection of the eukaryotic elongation factor 2 (eEF2) to ribosomes (Stickel et al., 2015). In alignment with these findings, our study reveals that YIV-818-A, RA-V, or RA-VII are also capable of inhibiting AR and CycD1 protein synthesis. This downregulation of AR and CycD1 proteins seems to be predominantly driven by the inhibition of protein synthesis rather than mRNA regulation. Taking into consideration the half-lives of AR and GR proteins - approximately 3 h (Syms et al., 1985) and 23 h (Dong et al., 1988) respectively, the pronounced AR protein downregulation following treatment with YIV-818-A, RA-V, or RA-VII can potentially be explained. It is plausible that the shorter half-life of AR proteins facilitates more noticeable effects from the treatments, as compared to GR proteins.

Interestingly, the inhibition of glucocorticoid receptor (GR) activity by YIV-818-A, RA-V, or RA-VII does not appear to directly correspond with protein downregulation. This led us to hypothesize that these compounds might be influencing the cofactors associated with the GR protein. Supporting this premise, our findings revealed that the levels of bromodomain proteins (Brd) and histone 3 lysine 27 acetylation (H3K27ac) were attenuated by YIV-818, RA-V, and RA-VII. Considering the pivotal role of Brd and H3K27ac in AR and GR-dependent enhancer activation and target gene transcription (Asangani et al., 2014; Nagarajan et al., 2014; Shah et al., 2017), this might represent one potential mechanism by which YIV-818-A, RA-V, or RA-VII inhibit GR activity. As part of future research, the utilization of a ChIP sequencing assay could provide valuable insights and further validate our hypothesis, determining if YIV-818A, RA-V, and RA-VII indeed affect GR activity by diminishing Brd and H3K27ac levels.

Notably, YIV-818-A, RA-V, or RA-VII have all exhibited a synergistic effect with enzalutamide, apalutamide, or darolutamide in the inhibition of AR activity, both in cell culture and *in vivo* (using the YIV-818 and enzalutamide combination in 22RV1, CRPC cells). By virtue of their ability to downregulate AR-V (AR splice variants) and hinder the GR activity of CRPC cells, the combination of these compounds with enzalutamide, apalutamide, or darolutamide might considerably decrease the chance of drug resistance development. Moreover, a potential reduction in drug dosage could possibly mitigate adverse effects experienced by patients. Therefore, YIV-818-A, RA-V, or RA-VII represent a promising approach to augment the therapeutic impact of enzalutamide, apalutamide, and darolutamide.

Furthermore, palbociclib, abemaciclib, and ribociclib, which are CDK4/6 inhibitors, are approved for the treatment of metastatic CRPC (Kase et al., 2020). Cyclin D1 (CycD1) serves as an activator for CDK4/6 kinases (Morgan, 1997). Upon the loss of CycD1, CDK4/6 kinases become inactive, thus unable to phosphorylate Rb (Bertoli et al., 2013). This hypophosphorylated Rb represses E2F, resulting in cell cycle progression arrest (Dick and Rubin, 2013). Since YIV-818-A, RA-V, or RA-VII downregulate CycD1, they may mirror the action of palbociclib, abemaciclib, and ribociclib in halting cell growth. Therefore, the potential application of YIV-818-A, RA-V, or RA-VII in the treatment of metastatic CRPC warrants further investigation.

At least twenty-four distinct bicyclic hexapeptides have been discovered and isolated from *Rubia cordifolia* (R.C.) (Shan et al., 2016). Existing research suggests that some of these bicyclic hexapeptides exhibit cytotoxic effects against tumor cells, P-388 leukemia cells, SGC-7901 human gastric adenocarcinoma cells, A-549 human non-small cell lung carcinoma cells, as well as HeLa (human cervical carcinoma) and KB (human epithelial carcinoma) cells (Itokawa et al., 1984; Lee et al., 2008a; Zhao et al., 2011). Moreover, a plethora of new bicyclic hexapeptides have been synthesized with an aim to enhance their anti-tumor activity (Lee et al., 2008a; Lee et al., 2008b; Hitotsuyanagi et al., 2011; Keysar et al., 2020).

Our study underlines the varying potency of different bicyclic hexapeptides, such as RA-V, RA-VII, or RA-XI, in inhibiting AR and GR. It is intriguing to note how a seemingly minor change, such as substituting glutamic acid at the 2nd position of the hexapeptide (as in RA-XI) instead of alanine (as in RA-V or RA-VII), can significantly reduce the anti-AR and anti-GR activity of RA-XI. This could be attributed to the bulky carboxylic acid group of glutamic acid potentially impeding its binding to the target molecule. On the other hand, the presence of a CH3 group on the tyrosine at position 6 in RA-VII appears to enhance its anti-AR and anti-GR activity compared to RA-V, which has an H group at the same position. The region spanning the 2nd to 6th positions could dictate the hexapeptide’s orientation towards its target, but this hypothesis warrants further investigation. A comprehensive study exploring the structure-activity relationship of this class of compounds in relation to AR should be pursued further. Such efforts could pave the way for the identification of more potent compounds with high selectivity towards AR, GR, or certain epigenetic states.

In addition to AR, AR-V (AR splice variants), and GR, prostate cancer progression and drug resistance involve a myriad of alternative mechanisms that have been extensively studied (Vander Ark et al., 2018). Notably, ACSL4 (acyl-CoA synthetase long-chain family member 4) has emerged as a significant player, as its high expression has been linked to promoting prostate cancer growth, invasion, and hormonal resistance, thereby contributing to disease aggressiveness (Wu et al., 2015). Another critical factor is AKR1C3, which plays a pivotal role in androgen-related pathways. This enzyme is responsible for metabolizing androstenediones into more-active androgens like testosterone or dihydrotestosterone, influencing androgen signaling in prostate cancer cells (Liu et al., 2015). Moreover, uncontrolled IL6/JAK/STAT3 signaling has been implicated in enzalutamide resistance. This pathway can transactivate androgen signaling, leading to reduced responsiveness to antiandrogen therapies like enzalutamide (Handle et al., 2016). Additionally, the transcription factor SOX2 has been found to promote lineage plasticity and antiandrogen resistance in prostate cancer cases lacking TP53 and RB1 tumor suppressor genes, contributing to the development of more aggressive and enzalutamide resistant phenotypes (Mu et al., 2017). Understanding these diverse mechanisms and their impact on prostate cancer progression is crucial for developing more effective therapeutic approaches. Exploring how YIV-818A, RA-V, and RA-VII interact with these targets can provide valuable insights into their potential as treatment options for prostate cancer, especially in cases with multiple resistance mechanisms. Targeting these alternative pathways could open new avenues for personalized and combination therapies, ultimately leading to improved outcomes for prostate cancer patients.

*Rubia cordifolia* (R.C) has a long-standing history of safe usage in Asia as a dietary supplement designed to improve health. *Rubia cordifolia* (R.C.) has been recognized for its diverse biological properties, including promoting coagulation, modulating the immune system, providing anti-inflammatory and neuroprotective effects, acting as an antioxidant, and exhibiting anti-tumor properties (Shan et al., 2016). However, its potential impact on androgen receptor (AR) and glucocorticoid receptor (GR) signaling in the treatment of prostate cancer has been largely overlooked. Our research presents compelling evidence to suggest that *Rubia cordifolia* (R.C.) may inhibit the growth of prostate cancer by disrupting AR and GR signaling. Thus, *Rubia cordifolia* (R.C.) emerges as a potentially powerful therapeutic agent for prostate cancer treatment or as a chemopreventive measure. This promising prospect is deserving of further investigation and consideration in future research endeavors.

We identified RA-V, a component of YIV-818-A, as the agent responsible for inhibiting AR and GR. However, we observed considerable variation in the quantity of RA-V present in *Rubia cordifolia* (R.C.) across different batches. Interestingly, the amount of RA-V present in *Rubia cordifolia* (R.C.) showed a strong correlation with AR inhibition across different batches. This suggests that RA-V could serve as a chemical marker to select high-potency *Rubia cordifolia* (R.C.) batches for development in the treatment or prevention of prostate cancer.

In summary, YIV-818-A, RA-V, or RA-VII show potential in overcoming the drug resistance encountered in castration-resistant prostate cancer (CRPC) cells caused by AR variants and GR. This is achieved by down-regulating AR protein, inhibiting GR function, and influencing epigenetic regulation. Agents such as YIV-818-A, RA-V, and RA-VII could potentially enhance the therapeutic effect of FDA-approved androgen inhibitors—enzalutamide, apalutamide, or darolutamide—against castration-resistant prostate cancer (CRPC). Moreover, RA-V, found in *Rubia cordifolia* (R.C.), could serve as a chemical marker for quality control in the preparation of this herbal supplement. Given the longstanding safe usage of *Rubia cordifolia* (R.C.) in Asia as a dietary supplement for health improvement, YIV-818-A could potentially be developed as a chemoprevention agent and/or anti-cancer drug for the treatment of prostate cancer.

## Data Availability

The original contributions presented in the study are included in the article/Supplementary Material, further inquiries can be directed to the corresponding author.

## References

[B1] Antonarakis E. S. Lu C. Wang H. Luber B. Nakazawa M. Roeser J. C. (2014). AR-V7 and resistance to enzalutamide and abiraterone in prostate cancer. N. Engl. J. Med. 371, 1028–1038. 10.1056/NEJMoa1315815 25184630 PMC4201502

[B2] Arora V. K. Schenkein E. Murali R. Subudhi S. K. Wongvipat J. Balbas M. D. (2013). Glucocorticoid receptor confers resistance to antiandrogens by bypassing androgen receptor blockade. Cell 155, 1309–1322. 10.1016/j.cell.2013.11.012 24315100 PMC3932525

[B3] Asangani I. A. Dommeti V. L. Wang X. Malik R. Cieslik M. Yang R. (2014). Therapeutic targeting of BET bromodomain proteins in castration-resistant prostate cancer. Nature 510, 278–282. 10.1038/nature13229 24759320 PMC4075966

[B4] Bertoli C. Skotheim J. M. De Bruin R. A. (2013). Control of cell cycle transcription during G1 and S phases. Nat. Rev. Mol. Cell Biol. 14, 518–528. 10.1038/nrm3629 23877564 PMC4569015

[B5] Buchanan G. Greenberg N. M. Scher H. I. Harris J. M. Marshall V. R. Tilley W. D. (2001). Collocation of androgen receptor gene mutations in prostate cancer. Clin. Cancer Res. 7, 1273–1281. 11350894

[B6] Cattrini C. Caffo O. De Giorgi U. Mennitto A. Gennari A. Olmos D. (2022). Apalutamide, darolutamide and enzalutamide for nonmetastatic castration-resistant prostate cancer (nmCRPC): A critical review. Cancers (Basel) 14, 1792. 10.3390/cancers14071792 35406564 PMC8997634

[B7] Chan J. Khan S. N. Harvey I. Merrick W. Pelletier J. (2004). Eukaryotic protein synthesis inhibitors identified by comparison of cytotoxicity profiles. RNA 10, 528–543. 10.1261/rna.5200204 14970397 PMC1370947

[B8] Chou T. C. Talalay P. (1977). A simple generalized equation for the analysis of multiple inhibitions of Michaelis-Menten kinetic systems. J. Biol. Chem. 252, 6438–6442. 10.1016/s0021-9258(17)39978-7 893418

[B9] Dick F. A. Rubin S. M. (2013). Molecular mechanisms underlying RB protein function. Nat. Rev. Mol. Cell Biol. 14, 297–306. 10.1038/nrm3567 23594950 PMC4754300

[B10] Dong Y. Poellinger L. Gustafsson J. A. Okret S. (1988). Regulation of glucocorticoid receptor expression: Evidence for transcriptional and posttranslational mechanisms. Mol. Endocrinol. 2, 1256–1264. 10.1210/mend-2-12-1256 3216865

[B11] Gioeli D. Paschal B. M. (2012). Post-translational modification of the androgen receptor. Mol. Cell Endocrinol. 352, 70–78. 10.1016/j.mce.2011.07.004 21820033

[B12] Handle F. Erb H. H. Luef B. Hoefer J. Dietrich D. Parson W. (2016). SOCS3 modulates the response to enzalutamide and is regulated by androgen receptor signaling and CpG methylation in prostate cancer cells. Mol. Cancer Res. 14, 574–585. 10.1158/1541-7786.MCR-15-0495 27053681

[B13] Hitotsuyanagi Y. Lee J. E. Kato S. Kim I. H. Kohashi H. Fukaya H. (2011). Per-N-methylated analogues of an antitumor bicyclic hexapeptide RA-VII. Bioorg Med. Chem. 19, 2458–2463. 10.1016/j.bmc.2011.02.003 21382716

[B14] Itokawa H. Takeya K. Mihara K. Mori N. Hamanaka T. Sonobe T. (1983). Studies on the antitumor cyclic hexapeptides obtained from Rubiae radix. Chem. Pharm. Bull. (Tokyo) 31, 1424–1427. 10.1248/cpb.31.1424 6627519

[B15] Itokawa H. Takeya K. Mori N. Takanashi M. Yamamoto H. Sonobe T. (1984). Cell growth-inhibitory effects of derivatives of antitumor cyclic hexapeptide RA-V obtained from Rubiae radix (V). Gan 75, 929–936. 6510638

[B16] Kase A. M. Copland J. A. Iii Tan W. (2020). Novel therapeutic strategies for CDK4/6 inhibitors in metastatic castrate-resistant prostate cancer. Onco Targets Ther. 13, 10499–10513. 10.2147/OTT.S266085 33116629 PMC7576355

[B17] Keysar S. B. Gomes N. Miller B. Jackson B. C. Le P. N. Morton J. J. (2020). Inhibiting translation elongation with SVC112 suppresses cancer stem cells and inhibits growth in Head and Neck squamous carcinoma. Cancer Res. 80, 1183–1198. 10.1158/0008-5472.CAN-19-3232 31911553 PMC7056512

[B18] Koivisto P. Kononen J. Palmberg C. Tammela T. Hyytinen E. Isola J. (1997). Androgen receptor gene amplification: A possible molecular mechanism for androgen deprivation therapy failure in prostate cancer. Cancer Res. 57, 314–319. 9000575

[B19] Lee J. E. Hitotsuyanagi Y. Fukaya H. Kondo K. Takeya K. (2008a). New cytotoxic bicyclic hexapeptides, RA-XXIII and RA-XXIV, from Rubia cordifolia L. Chem. Pharm. Bull. (Tokyo) 56, 730–733. 10.1248/cpb.56.730 18451569

[B20] Lee J. E. Hitotsuyanagi Y. Nakagawa Y. Kato S. Fukaya H. Takeya K. (2008b). Design and synthesis of a bis(cycloisodityrosine) analogue of RA-VII, an antitumor bicyclic hexapeptide. Bioorg Med. Chem. Lett. 18, 6458–6461. 10.1016/j.bmcl.2008.10.064 18993061

[B21] Li Y. Chan S. C. Brand L. J. Hwang T. H. Silverstein K. A. Dehm S. M. (2013). Androgen receptor splice variants mediate enzalutamide resistance in castration-resistant prostate cancer cell lines. Cancer Res. 73, 483–489. 10.1158/0008-5472.CAN-12-3630 23117885 PMC3549016

[B22] Liu C. Lou W. Zhu Y. Yang J. C. Nadiminty N. Gaikwad N. W. (2015). Intracrine androgens and AKR1C3 activation confer resistance to enzalutamide in prostate cancer. Cancer Res. 75, 1413–1422. 10.1158/0008-5472.CAN-14-3080 25649766 PMC4383695

[B23] Lu J. Lonergan P. E. Nacusi L. P. Wang L. Schmidt L. J. Sun Z. (2015). The cistrome and gene signature of androgen receptor splice variants in castration resistant prostate cancer cells. J. Urol. 193, 690–698. 10.1016/j.juro.2014.08.043 25132238 PMC4411637

[B24] Mills I. G. (2014). Maintaining and reprogramming genomic androgen receptor activity in prostate cancer. Nat. Rev. Cancer 14, 187–198. 10.1038/nrc3678 24561445

[B25] Morgan D. O. (1997). Cyclin-dependent kinases: Engines, clocks, and microprocessors. Annu. Rev. Cell Dev. Biol. 13, 261–291. 10.1146/annurev.cellbio.13.1.261 9442875

[B26] Nagarajan S. Hossan T. Alawi M. Najafova Z. Indenbirken D. Bedi U. (2014). Bromodomain protein BRD4 is required for estrogen receptor-dependent enhancer activation and gene transcription. Cell Rep. 8, 460–469. 10.1016/j.celrep.2014.06.016 25017071 PMC4747248

[B27] Nakazawa M. Antonarakis E. S. Luo J. (2014). Androgen receptor splice variants in the era of enzalutamide and abiraterone. Horm. Cancer 5, 265–273. 10.1007/s12672-014-0190-1 25048254 PMC4167475

[B28] Pomerantz M. M. Li F. Takeda D. Y. Lenci R. Chonkar A. Chabot M. (2015). The androgen receptor cistrome is extensively reprogrammed in human prostate tumorigenesis. Nat. Genet. 47, 1346–1351. 10.1038/ng.3419 26457646 PMC4707683

[B29] Rice M. A. Malhotra S. V. Stoyanova T. (2019). Second-generation antiandrogens: From discovery to standard of Care in castration resistant prostate cancer. Front. Oncol. 9, 801. 10.3389/fonc.2019.00801 31555580 PMC6723105

[B30] Shah N. Wang P. Wongvipat J. Karthaus W. R. Abida W. Armenia J. (2017). Regulation of the glucocorticoid receptor via a BET-dependent enhancer drives antiandrogen resistance in prostate cancer. Elife 6, e27861. 10.7554/eLife.27861 28891793 PMC5593504

[B31] Shan M. Yu S. Yan H. Chen P. Zhang L. Ding A. (2016). A Review of the Botany, Phytochemistry, Pharmacology and Toxicology of Rubiae Radix et Rhizoma. Molecules 21 (12), 1747. 10.3390/molecules21121747 27999402 PMC6274022

[B32] Shiota M. Yokomizo A. Naito S. (2011). Increased androgen receptor transcription: A cause of castration-resistant prostate cancer and a possible therapeutic target. J. Mol. Endocrinol. 47, R25–R41. 10.1530/JME-11-0018 21504942

[B33] Sramkoski R. M. Pretlow T. G. 2nd Giaconia J. M. Pretlow T. P. Schwartz S. Sy M. S. (1999). A new human prostate carcinoma cell line, 22Rv1. Vitro Cell Dev Biol Anim 35, 403–409. 10.1007/s11626-999-0115-4 10462204

[B34] Stickel S. A. Gomes N. P. Frederick B. Raben D. Su T. T. (2015). Bouvardin is a radiation modulator with a novel mechanism of action. Radiat. Res. 184, 392–403. 10.1667/RR14068.1 26414509 PMC4643058

[B35] Syms A. J. Norris J. S. Panko W. B. Smith R. G. (1985). Mechanism of androgen-receptor augmentation. Analysis of receptor synthesis and degradation by the density-shift technique. J. Biol. Chem. 260, 455–461. 10.1016/s0021-9258(18)89753-8 3871197

[B36] Tan M. E. Li J. Xu H. E. Melcher K. Yong E. (2015). Androgen receptor: Structure, role in prostate cancer and drug discovery. Acta Pharmacol. Sin. 36, 3–23. 10.1038/aps.2014.18 24909511 PMC4571323

[B37] Vander Ark A. Cao J. Li X. (2018). Mechanisms and approaches for overcoming enzalutamide resistance in prostate cancer. Front. Oncol. 8, 180. 10.3389/fonc.2018.00180 29911070 PMC5992404

[B38] Wong Y. N. Ferraldeschi R. Attard G. De Bono J. (2014). Evolution of androgen receptor targeted therapy for advanced prostate cancer. Nat. Rev. Clin. Oncol. 11, 365–376. 10.1038/nrclinonc.2014.72 24840076

[B39] Wu X. Deng F. Li Y. Daniels G. Du X. Ren Q. (2015). ACSL4 promotes prostate cancer growth, invasion and hormonal resistance. Oncotarget 6, 44849–44863. 10.18632/oncotarget.6438 26636648 PMC4792596

[B40] Zalacain M. Zaera E. Vazquez D. Jimenez A. (1982). The mode of action of the antitumor drug bouvardin, an inhibitor of protein synthesis in eukaryotic cells. FEBS Lett. 148, 95–97. 10.1016/0014-5793(82)81250-7 6924616

[B41] Zhao S. M. Kuang B. Fan J. T. Yan H. Xu W. Y. Tan N. H. (2011). Antitumor cyclic hexapeptides from rubia plants: History, chemistry, and mechanism (2005-2011). Chim. (Aarau) 65, 952–956. 10.2533/chimia.2011.952 22273378

