# Supplementary material for: YIV-818-A: a novel therapeutic agent in prostate cancer management through androgen receptor downregulation, glucocorticoid receptor inhibition, epigenetic regulation, and enhancement of apalutamide, darolutamide, and enzalutamide efficacy
Source: Front Pharmacol. 2023 Oct 4;14:1244655. doi: 10.3389/fphar.2023.1244655 (PMC10582333; doi:10.3389/fphar.2023.1244655)
Supplement: Supplementary file 1 [file Presentation1.PPTX]

## Slide 1
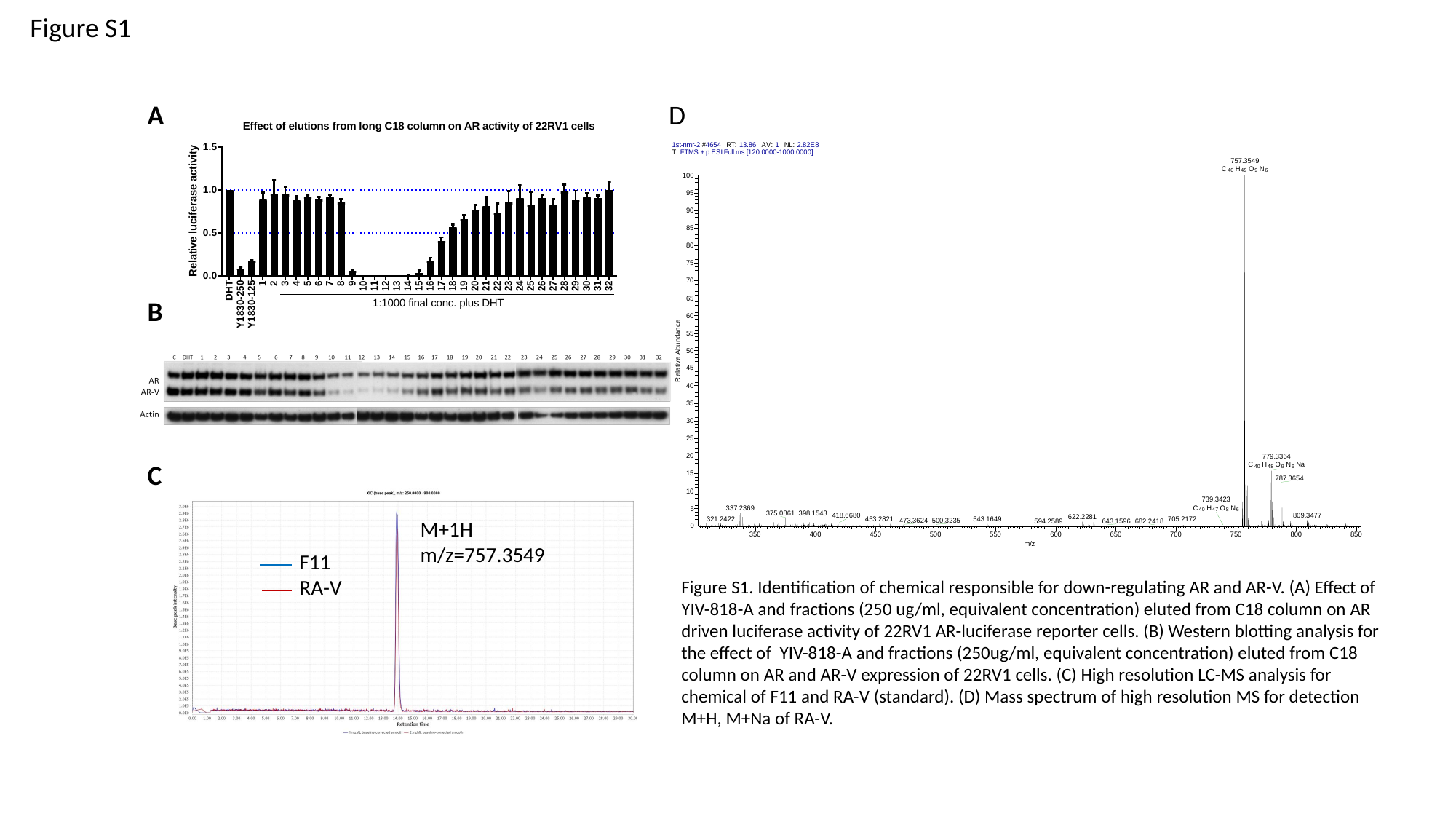

Figure S1
A
B
C
F11
RA-V
D
M+1H
m/z=757.3549
Figure S1. Identification of chemical responsible for down-regulating AR and AR-V. (A) Effect of YIV-818-A and fractions (250 ug/ml, equivalent concentration) eluted from C18 column on AR driven luciferase activity of 22RV1 AR-luciferase reporter cells. (B) Western blotting analysis for the effect of YIV-818-A and fractions (250ug/ml, equivalent concentration) eluted from C18 column on AR and AR-V expression of 22RV1 cells. (C) High resolution LC-MS analysis for chemical of F11 and RA-V (standard). (D) Mass spectrum of high resolution MS for detection M+H, M+Na of RA-V.

## Slide 2
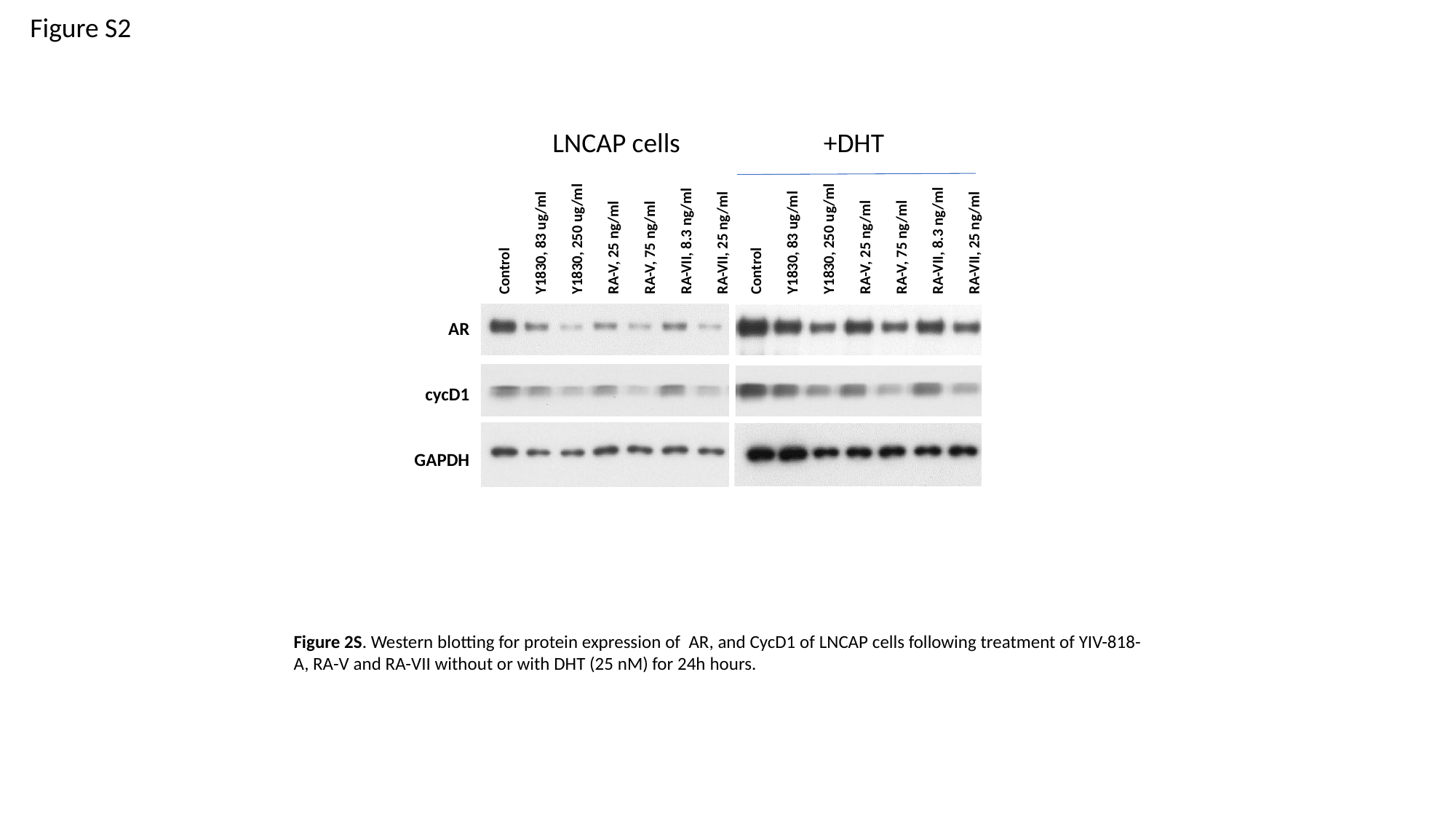

Figure S2
Control
Y1830, 83 ug/ml
Y1830, 250 ug/ml
RA-V, 25 ng/ml
RA-V, 75 ng/ml
RA-VII, 8.3 ng/ml
RA-VII, 25 ng/ml
Control
Y1830, 83 ug/ml
Y1830, 250 ug/ml
RA-V, 25 ng/ml
RA-V, 75 ng/ml
RA-VII, 8.3 ng/ml
RA-VII, 25 ng/ml
LNCAP cells
+DHT
AR
cycD1
GAPDH
Figure 2S. Western blotting for protein expression of AR, and CycD1 of LNCAP cells following treatment of YIV-818-A, RA-V and RA-VII without or with DHT (25 nM) for 24h hours.

## Slide 3
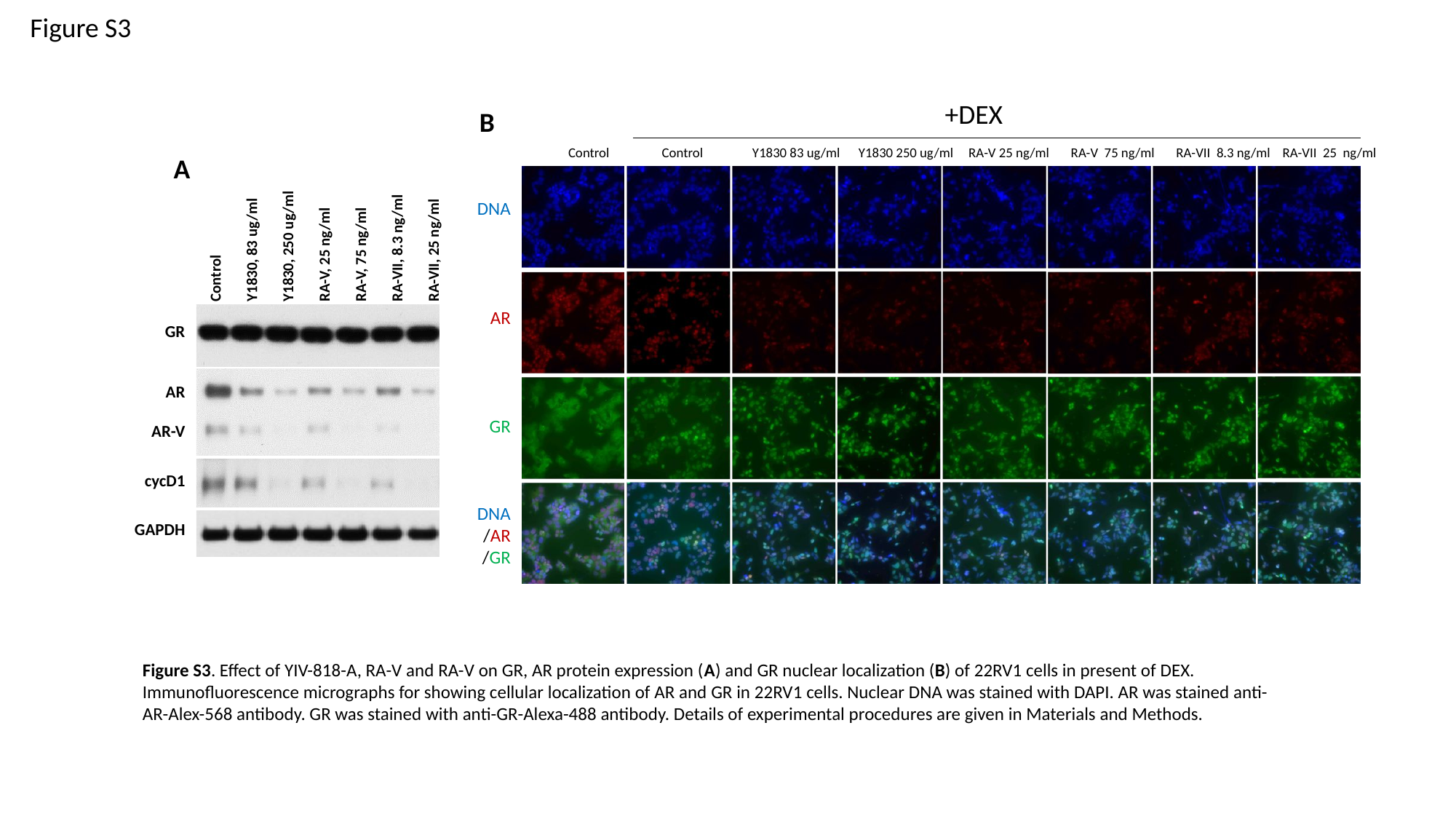

Figure S3
+DEX
B
Control Control Y1830 83 ug/ml Y1830 250 ug/ml RA-V 25 ng/ml RA-V 75 ng/ml RA-VII 8.3 ng/ml RA-VII 25 ng/ml
DNA
AR
GR
DNA
/AR
/GR
A
Control
Y1830, 83 ug/ml
Y1830, 250 ug/ml
RA-V, 25 ng/ml
RA-V, 75 ng/ml
RA-VII, 8.3 ng/ml
RA-VII, 25 ng/ml
GR
AR
AR-V
cycD1
GAPDH
Figure S3. Effect of YIV-818-A, RA-V and RA-V on GR, AR protein expression (A) and GR nuclear localization (B) of 22RV1 cells in present of DEX. Immunofluorescence micrographs for showing cellular localization of AR and GR in 22RV1 cells. Nuclear DNA was stained with DAPI. AR was stained anti-AR-Alex-568 antibody. GR was stained with anti-GR-Alexa-488 antibody. Details of experimental procedures are given in Materials and Methods.

## Slide 4
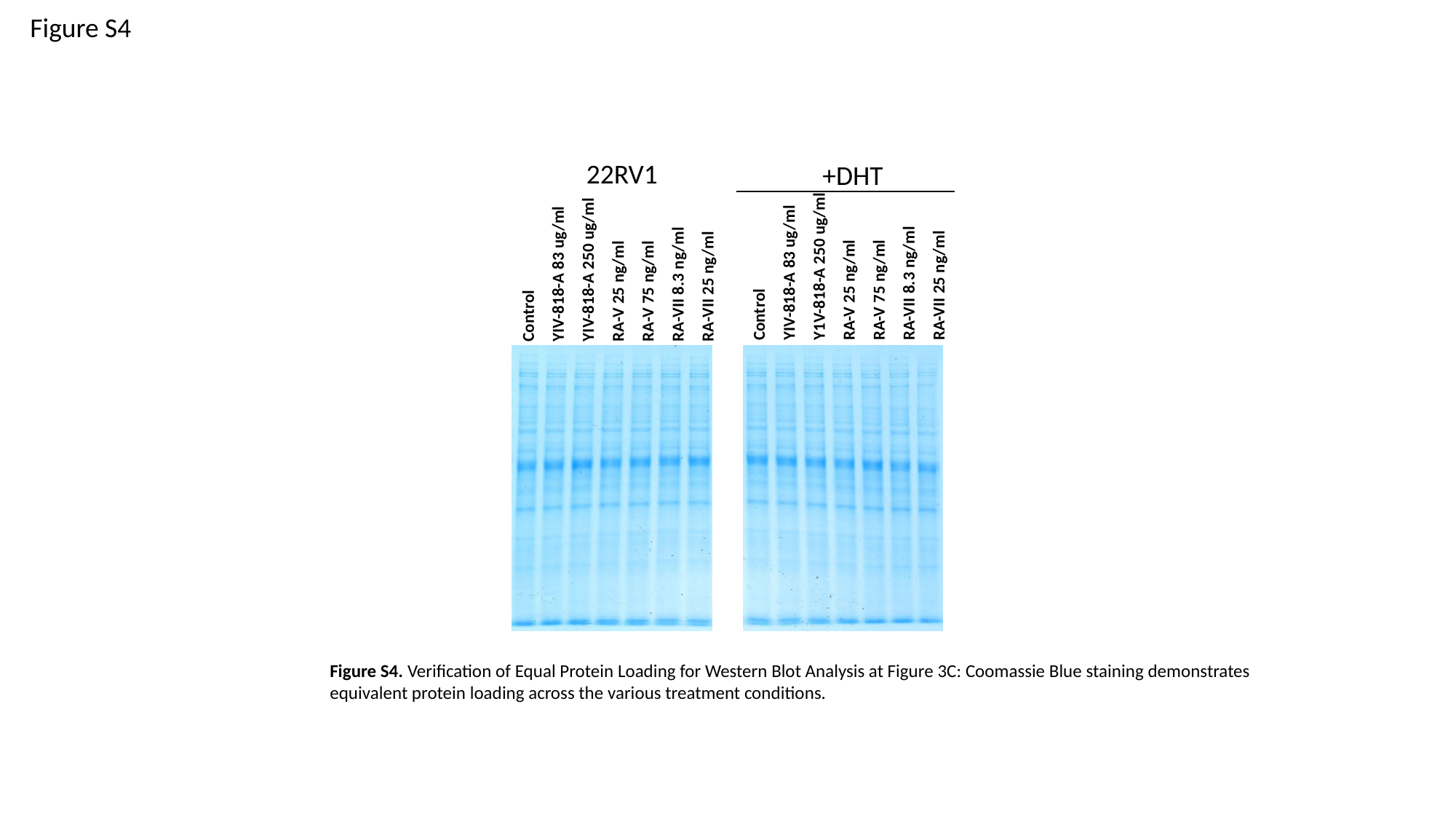

Figure S4
22RV1
+DHT
Control
YIV-818-A 83 ug/ml
Y1V-818-A 250 ug/ml
RA-V 25 ng/ml
RA-V 75 ng/ml
RA-VII 8.3 ng/ml
RA-VII 25 ng/ml
Control
YIV-818-A 83 ug/ml
YIV-818-A 250 ug/ml
RA-V 25 ng/ml
RA-V 75 ng/ml
RA-VII 8.3 ng/ml
RA-VII 25 ng/ml
Figure S4. Verification of Equal Protein Loading for Western Blot Analysis at Figure 3C: Coomassie Blue staining demonstrates equivalent protein loading across the various treatment conditions.
