# Supplementary material for: YIV-818-A: a novel therapeutic agent in prostate cancer management through androgen receptor downregulation, glucocorticoid receptor inhibition, epigenetic regulation, and enhancement of apalutamide, darolutamide, and enzalutamide efficacy
Source: Front Pharmacol. 2023 Oct 4;14:1244655. doi: 10.3389/fphar.2023.1244655 (PMC10582333; doi:10.3389/fphar.2023.1244655)
Supplement: Supplementary file 2 [file DataSheet1.docx]

**Supplementary data for NMR analysis**


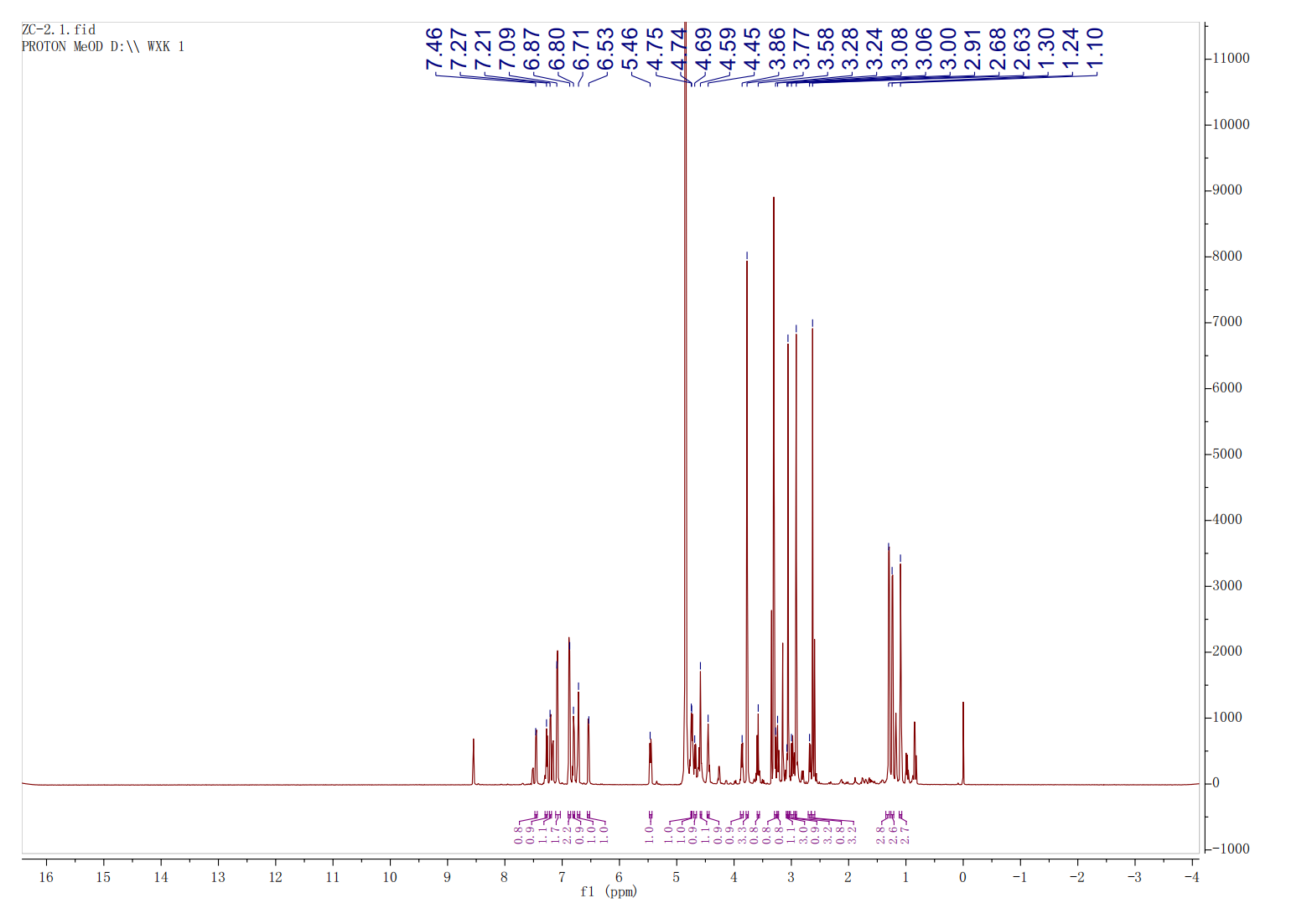


Figure 1. ^1^H spectrum of compound (MeOD)


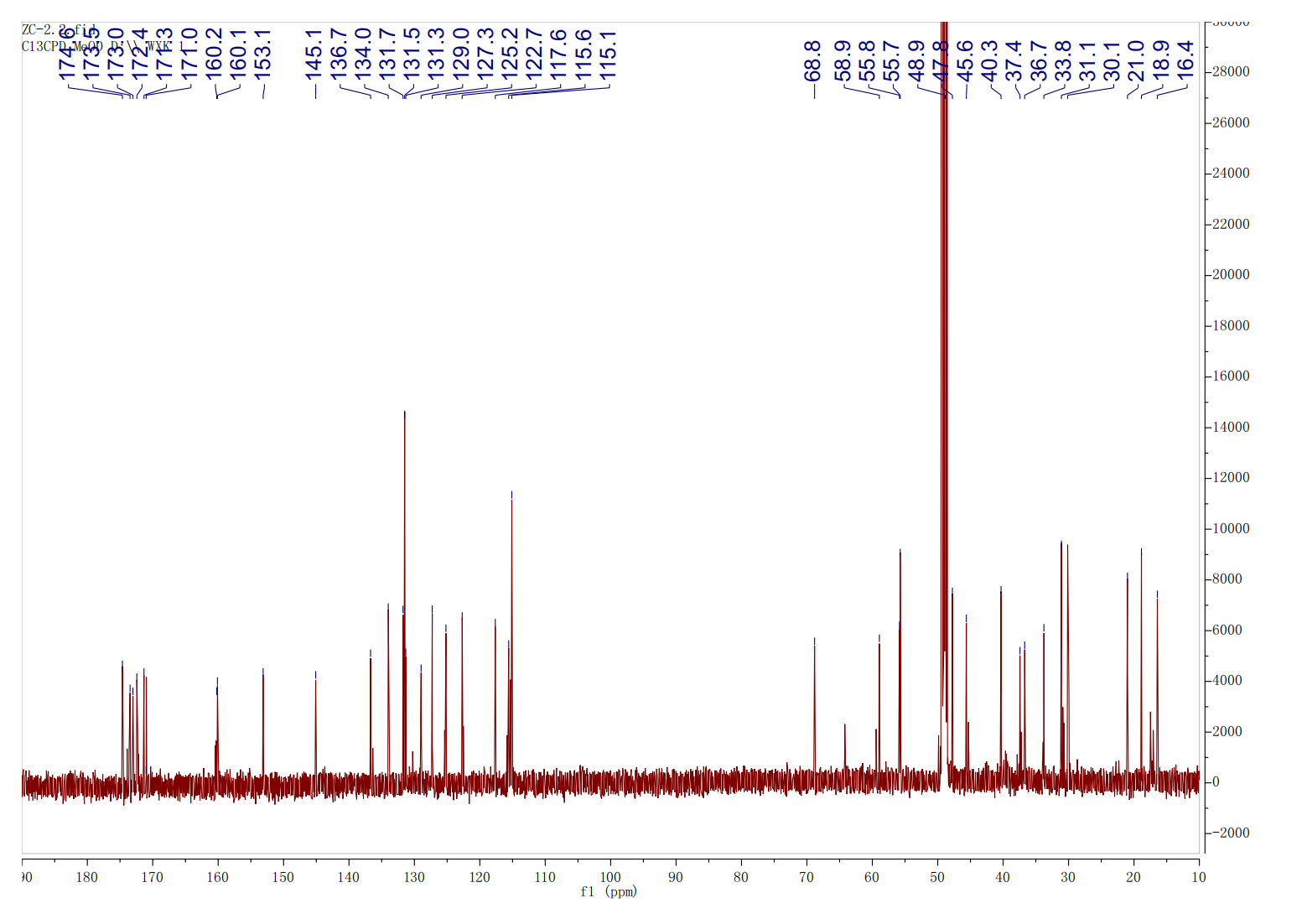


Figure 2. ^13^C spectrum of compound (MeOD)

**
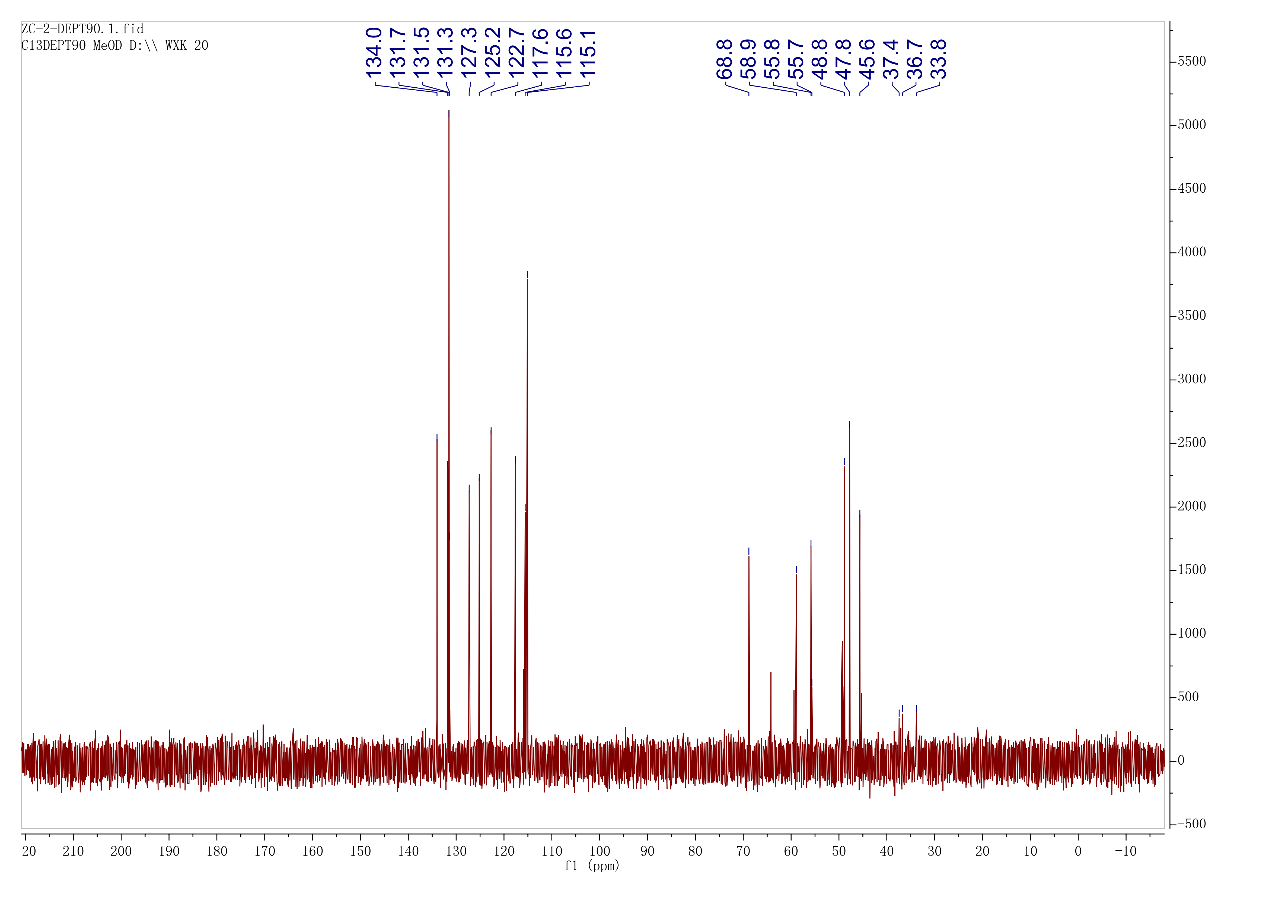
**Figure 3. DEPT 90 spectrum of compound (MeOD)

**
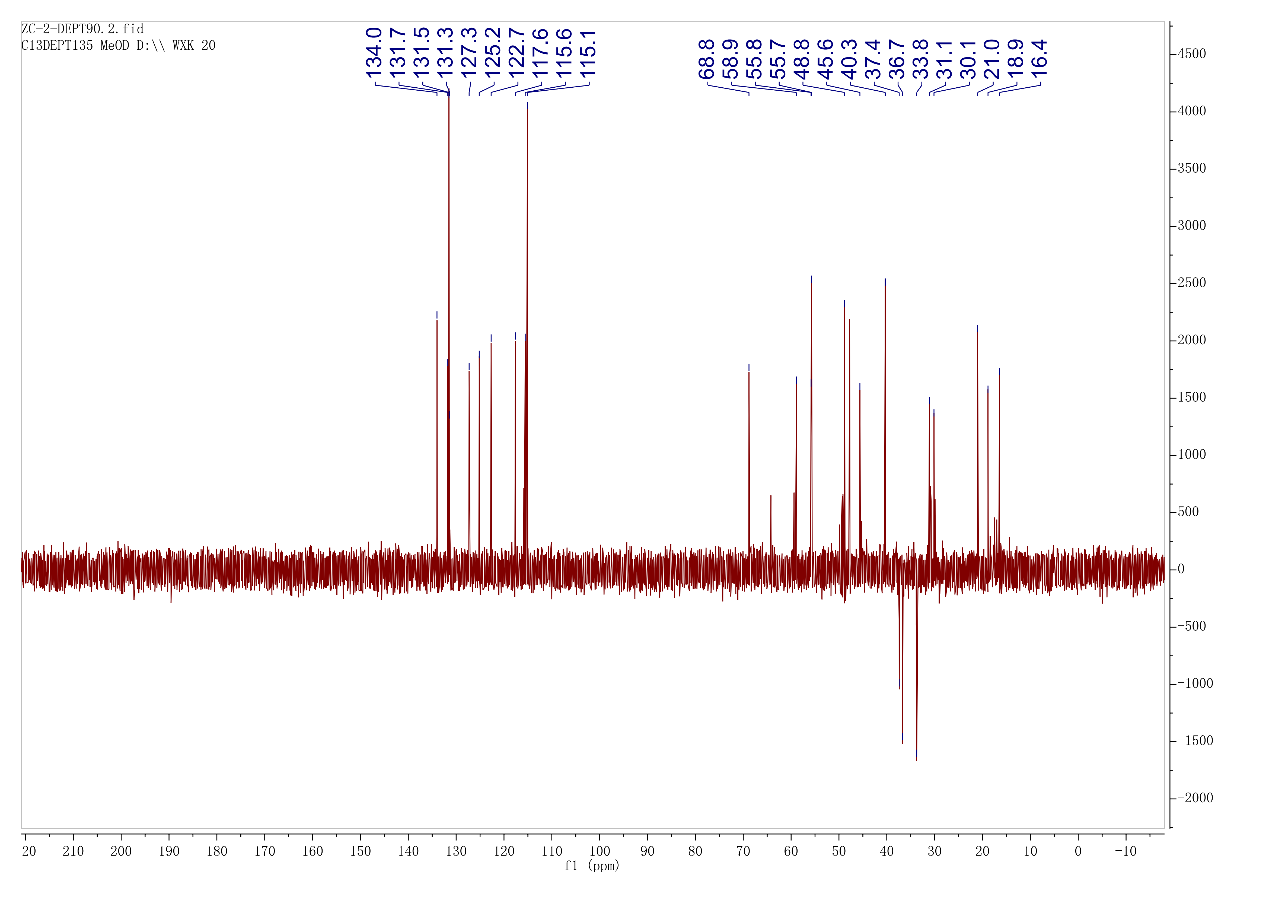
**

Figure 4. DEPT 135 spectrum of compound (MeOD)

**
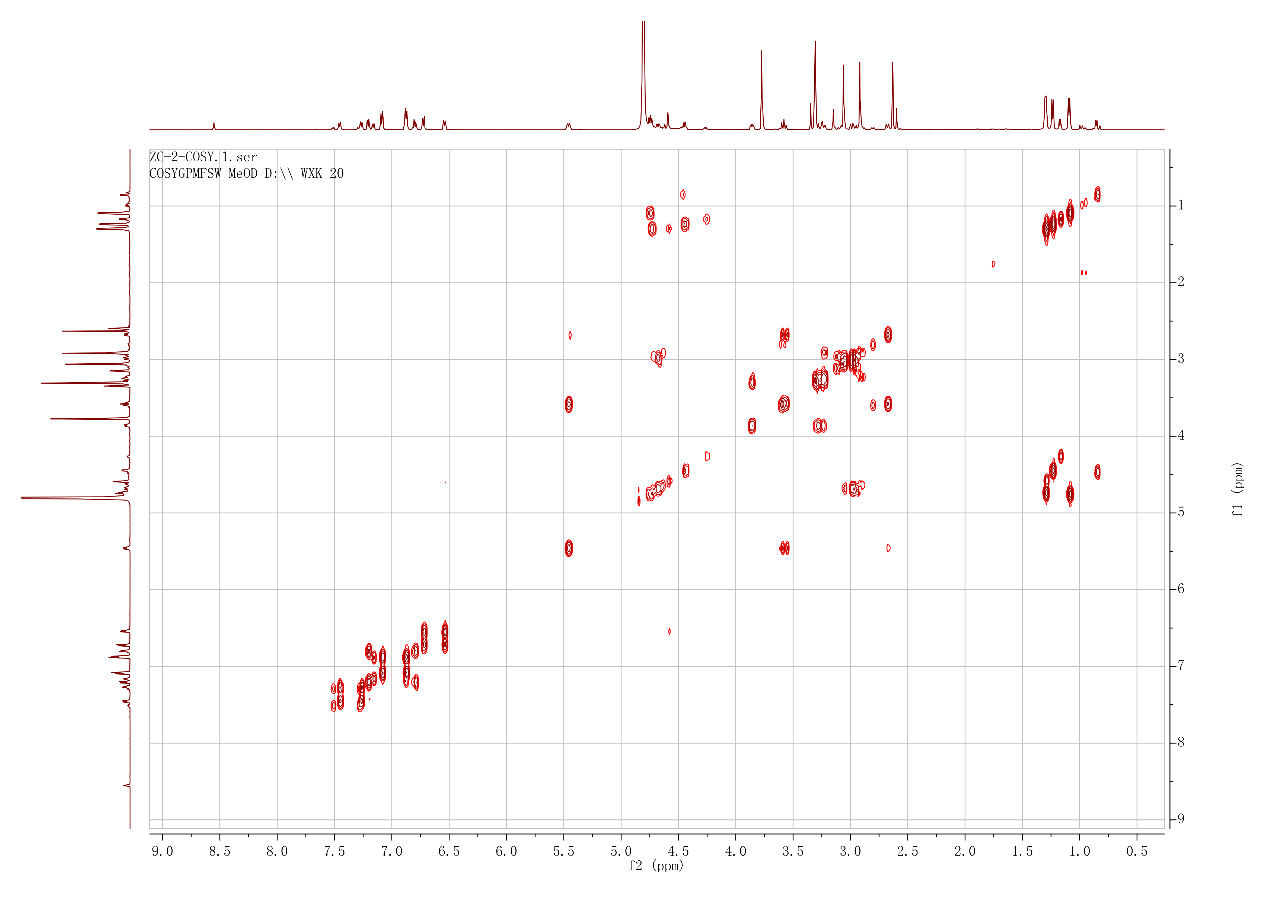
**

Figure 5. COSY spectrum of compound (MeOD)

**
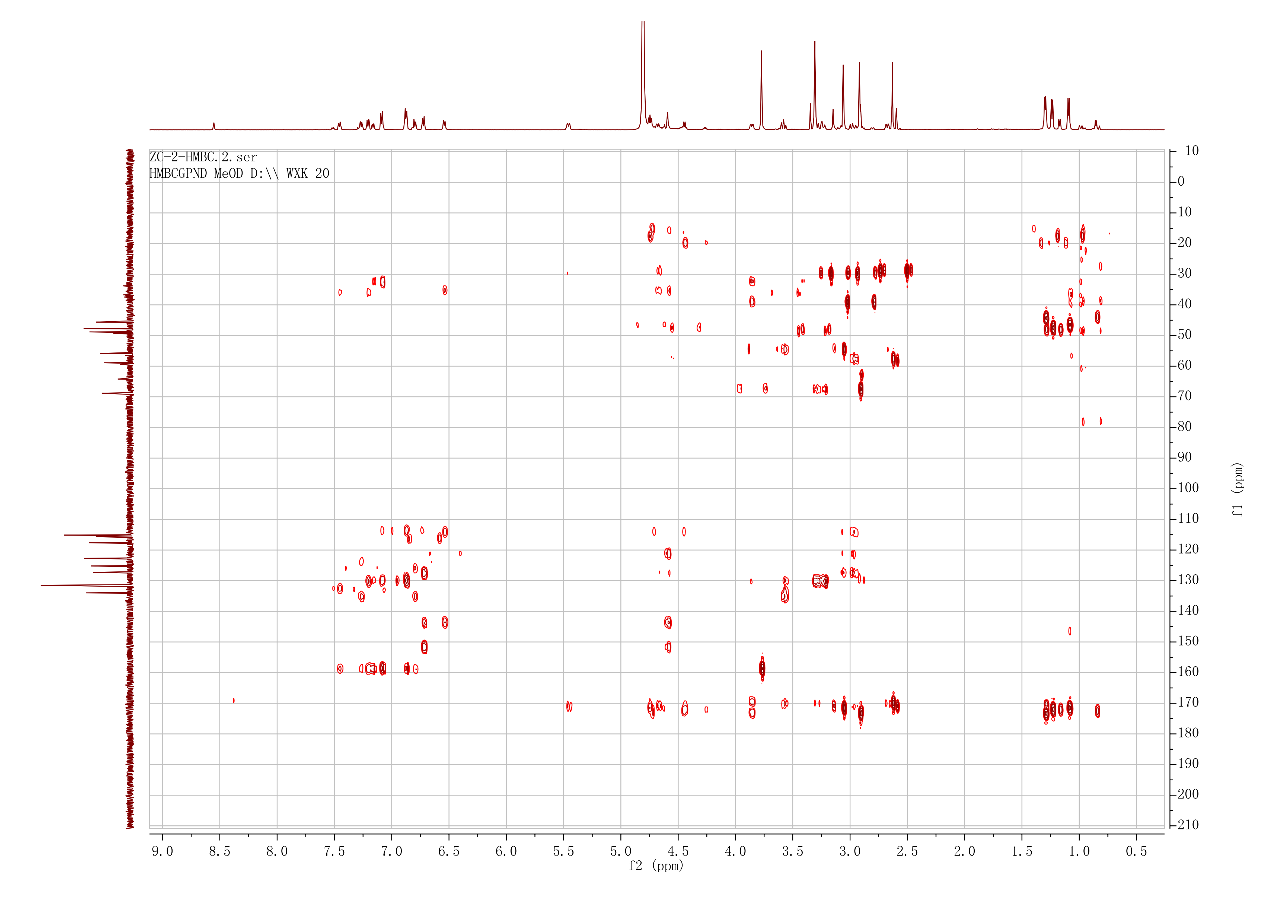
**

Figure 6. HMBC spectrum of compound (MeOD)

**
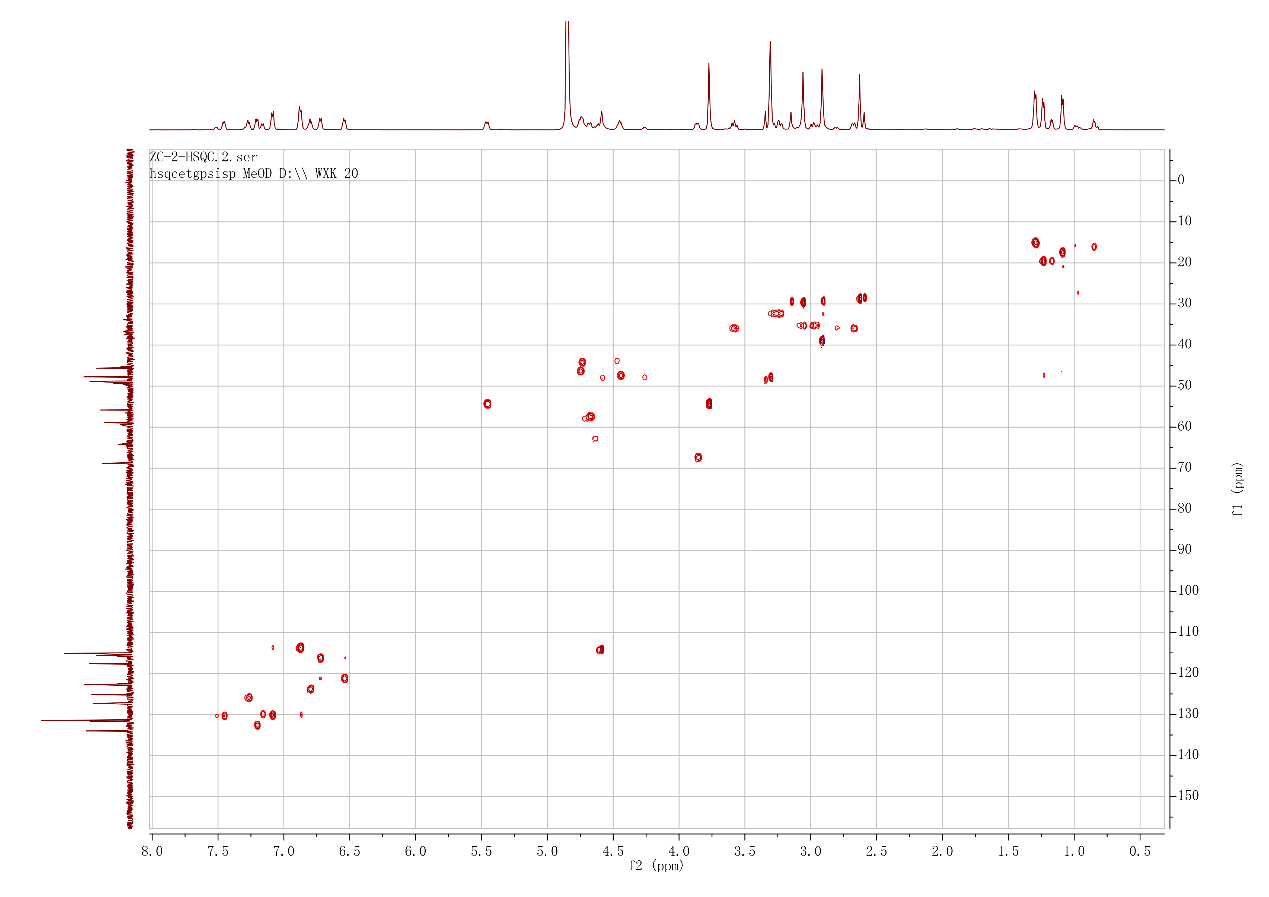
**Figure 7. HSQC spectrum of compound (MeOD)

**
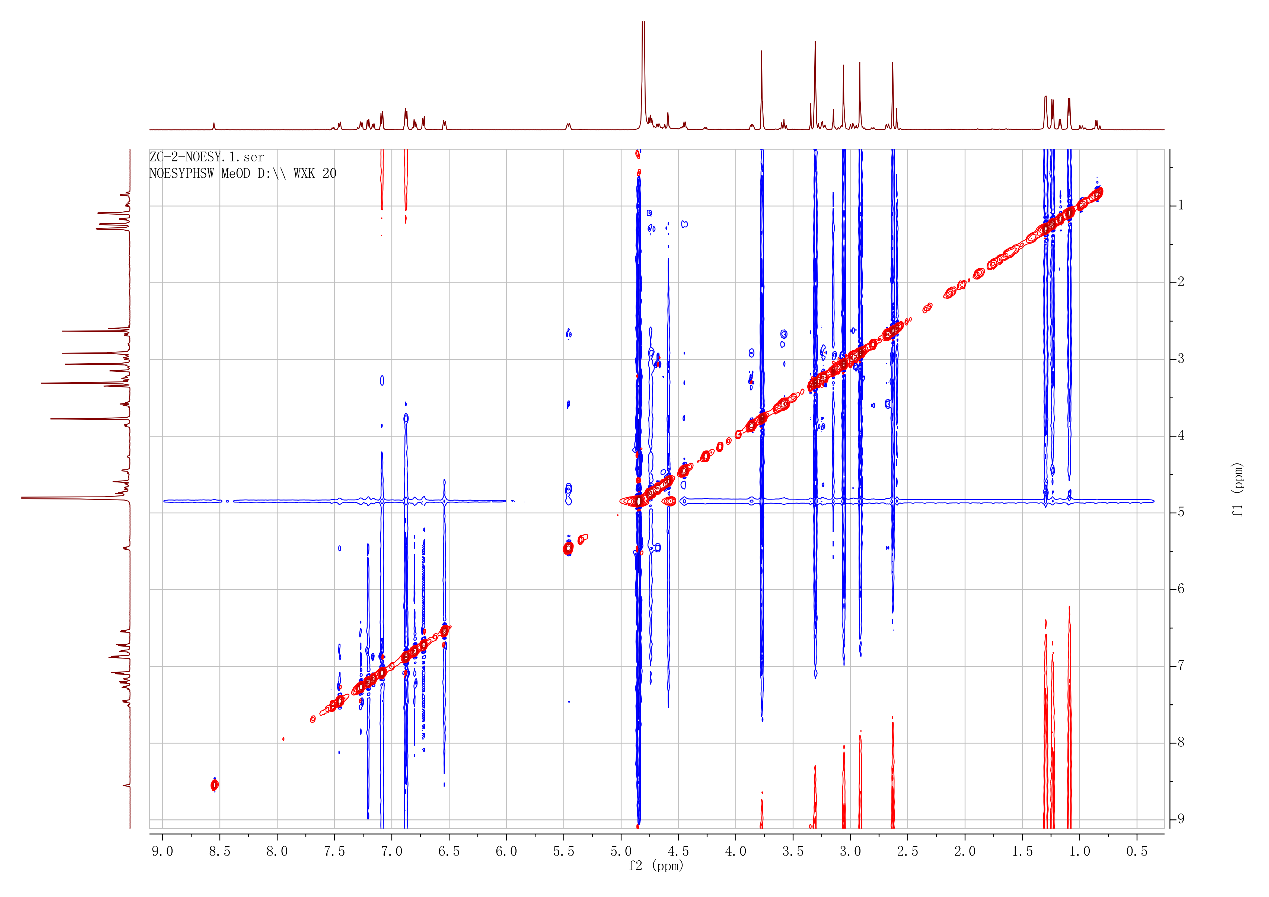
**

Figure 8. NOESY spectrum of compound (MeOD)

Figure 9. The structure of this compound

Table 1 the NMR data of the chemical of F11 (MeOD)

| No. | ^13^C | ^1^H | HHCOSY | HMBC |
| --- | --- | --- | --- | --- |
| 1 | 45.6 | 4.74 (dt, 11.8, 7.0, 1H) | 1-CH_3_ | C-1-CH_3_/2 |
| 2 | 174.6 |  |  |  |
| 3-N-CH_3_ | 40.3 | 2.91 (s, 3H) |  | 2, 4 |
| 4 | 68.8 | 3.86 (dd, 11.0, 5.0, 1H) | 19 | 19, 20, 5 |
| 5 | 171.0 |  |  |  |
| 7 | 47.8 | 4.75 (m, 1H) | 7-CH_3_ | 7-CH_3_/8 |
| 8 | 173.0 |  |  |  |
| 9-N-CH_3_ | 31.1 | 3.06 (s, 3H) |  | C-10/8 |
| 10 | 55.8 | 5.46 (dd, 11.5, 3.5, 1H) | 24 | 11, 25 |
| 11 | 171.3 |  |  |  |
| 12-N-CH_3_ | 30.1 | 2.63 (s, 3H) |  | 11, 13 |
| 13 | 58.9 | 4.69 (dd, 11.8, 3.9, 1H) | 31 | 14, 31, 32 |
| 14 | 172.4 |  |  |  |
| 16 | 48.9 | 4.45 (d, 7.0 Hz, 1H) | 16-CH_3_ | 16-CH_3_/17 |
| 17 | 173.5 |  |  |  |
| 19 | 33.8 | 3.28 (d, 12.5, 1H)  3.24 (dd, 14.0 , 4.7, 1H) |  | 4, 21 |
| 20 | 131.3 |  |  |  |
| 21 | 131.5×2 | 7.09 (d, 8.5, 2H) | 22 | 19, 20, 23 |
| 22 | 115.1×2 | 6.87 (d, 8.5, 2H) | 21 | 20, 21, 23 |
| 23 | 160.2 |  |  |  |
| 24 | 37.4 | 2.68 (dd, 11.5, 3.1, 1H)  3.58 (t, 11.5, 1H) | 10 | 11, 10, 26, 30 |
| 25 | 136.7 |  |  |  |
| 26 | 131.7 | 7.46 (dd, 8.5, 2.2, 1H) | 27 | 28, 30 |
| 27 | 127.3 | 7.27 (dd, 8.4, 2.4, 1H) | 26 | 25, 29 |
| 28 | 160.1 |  |  |  |
| 29 | 125.2 | 6.80 (dd, 8.5, 2.4, 1H) | 30 | 25, 27 |
| 30 | 134.0 | 7.21 (dd, 8.5, 2.2, 1H) | 29 | 26, 28 |
| 31 | 36.7 | 3.08 (dd, 12,1, 3.9, 1H)  3.00 (d, 12.1, 1H) | 13 | 14, 33, 37 |
| 32 | 129.0 |  |  |  |
| 33 | 115.6 | 4.59 (d, 2.2 Hz, 1H) |  | 31, 35, 37 |
| 34 | 153.1 |  |  |  |
| 35 | 145.1 |  |  |  |
| 36 | 117.6 | 6.71 (d, 8.2, 1H) | 37 | 32, 34 |
| 37 | 122.7 | 6.53 (dd, 8.3, 2.2, 1H) | 36 | 33, 35 |
| 1-CH_3_ | 16.4 | 1.30 (d, 6.9, 3H) | 1 | 1, 2 |
| 7-CH_3_ | 18.9 | 1.10 (d, 6.7, 3H) | 7 | 7, 8 |
| 16-CH_3_ | 21.0 | 1.24 (d, 7.0, 3H) | 16 | 16, 17 |
| 23-O-CH_3_ | 55.7 | 3.77 (s, 3H) |  | 23 |


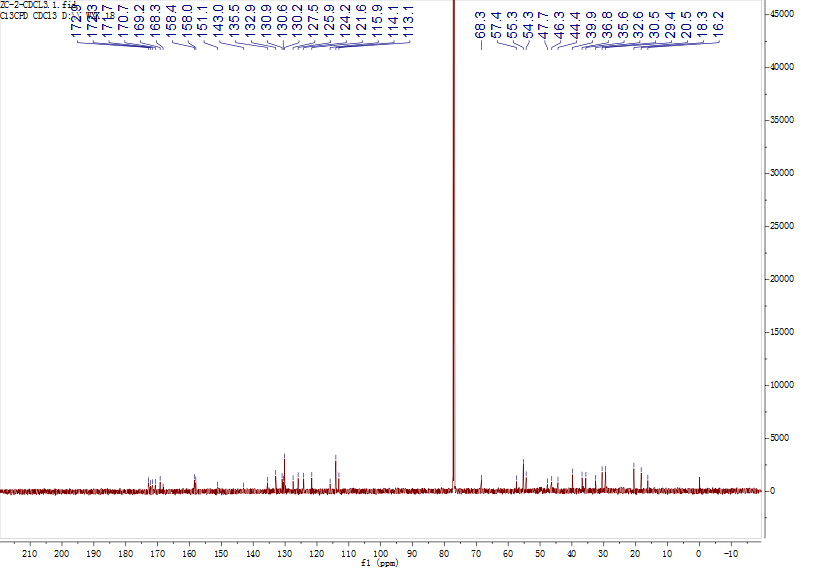


Figure 10 ^13^C spectrum of compound (CDCl3)
